# metadeconfoundR: Covariate analysis of high-dimensional cross-sectional omics data

**DOI:** 10.1093/bioadv/vbag242

**Published:** 2026-08-19

**Authors:** Till Birkner, Chia-Yu Chen, Morgan Essex, Kilian Dahm, Ulrike Löber, Thomas Ulas, Víctor Hugo Jarquín-Díaz, Sofia Kirke Forslund-Startceva

**Affiliations:** Max-Delbrück Center for Molecular Medicine in the Helmholtz Association (MDC), Berlin, Germany; Experimental and Clinical Research Center (ECRC), a cooperation of the Max-Delbrück Center and Charité-Universitätsmedizin Berlin, Berlin, Germany; Charité-Universitätsmedizin Berlin, a corporate member of Freie Universität Berlin and Humboldt-Universität zu Berlin, Berlin, Germany; German Center for Cardiovascular Research (DZHK), Berlin, Germany; Max-Delbrück Center for Molecular Medicine in the Helmholtz Association (MDC), Berlin, Germany; Experimental and Clinical Research Center (ECRC), a cooperation of the Max-Delbrück Center and Charité-Universitätsmedizin Berlin, Berlin, Germany; Charité-Universitätsmedizin Berlin, a corporate member of Freie Universität Berlin and Humboldt-Universität zu Berlin, Berlin, Germany; Max-Delbrück Center for Molecular Medicine in the Helmholtz Association (MDC), Berlin, Germany; Experimental and Clinical Research Center (ECRC), a cooperation of the Max-Delbrück Center and Charité-Universitätsmedizin Berlin, Berlin, Germany; Charité-Universitätsmedizin Berlin, a corporate member of Freie Universität Berlin and Humboldt-Universität zu Berlin, Berlin, Germany; Systems Medicine, German Center for Neurodegenerative Diseases (DZNE), Bonn, Germany; Genomics and Immunoregulation, Life & Medical Sciences (LIMES) Institute, University of Bonn, Bonn, Germany; Max-Delbrück Center for Molecular Medicine in the Helmholtz Association (MDC), Berlin, Germany; Experimental and Clinical Research Center (ECRC), a cooperation of the Max-Delbrück Center and Charité-Universitätsmedizin Berlin, Berlin, Germany; Charité-Universitätsmedizin Berlin, a corporate member of Freie Universität Berlin and Humboldt-Universität zu Berlin, Berlin, Germany; German Center for Cardiovascular Research (DZHK), Berlin, Germany; Systems Medicine, German Center for Neurodegenerative Diseases (DZNE), Bonn, Germany; Genomics and Immunoregulation, Life & Medical Sciences (LIMES) Institute, University of Bonn, Bonn, Germany; Platform for Single Cell Genomics and Epigenomics, German Center for Neurodegenerative Diseases (DZNE), the University of Bonn and Western German Genome Center (WGGC), Bonn, Germany; Max-Delbrück Center for Molecular Medicine in the Helmholtz Association (MDC), Berlin, Germany; Experimental and Clinical Research Center (ECRC), a cooperation of the Max-Delbrück Center and Charité-Universitätsmedizin Berlin, Berlin, Germany; Charité-Universitätsmedizin Berlin, a corporate member of Freie Universität Berlin and Humboldt-Universität zu Berlin, Berlin, Germany; Max-Delbrück Center for Molecular Medicine in the Helmholtz Association (MDC), Berlin, Germany; Experimental and Clinical Research Center (ECRC), a cooperation of the Max-Delbrück Center and Charité-Universitätsmedizin Berlin, Berlin, Germany; Charité-Universitätsmedizin Berlin, a corporate member of Freie Universität Berlin and Humboldt-Universität zu Berlin, Berlin, Germany; German Center for Cardiovascular Research (DZHK), Berlin, Germany; Structural and Computational Biology Unit (SCB), EMBL Heidelberg, Heidelberg, Germany

## Abstract

**Motivation:**

Identifying disease biomarkers from large molecular datasets is complicated by correlated and confounded signals like comorbidities and treatment regimens, batch effects, and cohort biases. These effects bias statistical inference and clinical conclusions. Robust methodologies are fundamental for reliable biomarker discovery.

**Results:**

metadeconfoundR is an R package for conservative biomarker discovery in (multi-)omics case-control datasets. It has a scalable two-step confounder-aware statistical framework for retaining only associations with independent support. It identifies covariate-naive univariate associations between omics features and metadata, then re-evaluates these associations using parallel post-hoc nested linear model testing to account for potential confounders. Confounded associations are flagged if they fully reduce to at least one other variable. metadeconfoundR supports parallel computation for large-scale datasets, offers visualization and tools for interpreting results and secondary analyses. We benchmark metadeconfoundR against state-of-the-art methods for identifying biomarkers using simulated ground truth derived from microbiome data, and demonstrate its ability to disentangle confounding effects while preserving statistical power, offering particular advantage when multiple covariates are present. metadeconfoundR functions for any -omics data type with continuous or categorical metadata/covariates.

**Availability:**

metadeconfoundR is available on CRAN (https://cran.r-project.org/web/packages/metadeconfoundR/) and GitHub (https://github.com/TillBirkner/metadeconfoundR).

## 1 Introduction

Advancements in several high-throughput omics technologies have exponentially expanded the availability of high dimensional biological data. While this enables the application of new systems medicine approaches to understand complex medical conditions, appropriate computational tools capable of efficiently processing these large quantities of data need to be developed. The higher complexity in the datasets increases the risk of underestimating confounding effects, which if unaccounted for may result in false (or rather incorrectly attributed) conclusions despite apparent statistical significance. In biomedical research using patient or population cohort data, covariates like medication and comorbid conditions often differ systematically between healthy and disease populations, which can introduce considerable biases that mask or falsely enhance associations between biomarkers and disease states. For example, both short- and long-term use of antibiotics can substantially alter the gut microbiome and metabolome, potentially confounding associations with disease (Korpela *et al.* 2016, Palleja *et al.* 2018, Forslund *et al.* 2021). A more concrete case involves type 2 diabetes, where previously contradictory microbiome associations could only be resolved after accounting for treatment with antidiabetic medication (Forslund *et al.* 2015). Other examples come from reverse causation, where, for example, certain risk factors may result in so severe illness that carriers are absent or fall under exclusion criteria for the studied patient population, leading to a situation where these factors paradoxically are more prevalent in the control population despite being causally linked to disease progression.

Besides the biological confounding effects, technical variation can introduce broader biases when integrating multiple batches or datasets, frequently unavoidable for practical reasons for larger cohorts. Differences in DNA extraction protocols can, for example, introduce effects equal or greater to the biological variation (Bartolomaeus *et al.* 2021).

In many cases, careful and detailed follow-up, especially through validation experiments, can help resolve such cases. In addition, a range of design-level strategies is commonly applied to mitigate confounding effects, including sample matching, stratification, or the exclusion of individuals with specific comorbidities or treatments. While such approaches can reduce known sources of bias, they are often limited by reduced sample sizes, incomplete covariate information, or the inability to account for multiple, partially correlated covariates simultaneously. Realistically, however, to not merely reproduce already established knowledge, such investment of effort requires a prior shortlist of possible biological relationships, the generation of which is the main purpose of the hypothesis-generating and untargeted biomarker- and mechanism-mining approaches that have emerged over the last two decades alongside advances in high-throughput technologies and computational capacity. Accordingly, there is a need for a “first-line” covariate management strategy which can be automated and thus integrated into workflows that conduct a large number of parallel statistical tests.

Here, we developed the R package metadeconfoundR, a statistical framework specifically designed to identify and mitigate confounding effects in cross-sectional case-control studies aiming to identify associations accurately attributed to a particular independent variable such as a disease state, rather than to any of an arbitrary number of simultaneously tracked potential covariates. This package implements a two-step approach that first detects “naive” (assuming no confounding influence) univariate associations and subsequently applies post-hoc nested model comparisons to determine whether detected associations are confounded, here defined as being fully statistically reducible to one or more of the covariates. MetadeconfoundR systematically evaluates whether an observed association remains significant after adjusting for other variables by using likelihood ratio tests (LRTs) between individual nested models. These tests are applied as a set of parallel post-hoc tests acting as a “filter” for the initial association. As a result, maximal model complexity evaluated does not grow with the number of considered covariates, avoiding instability of multivariate models where variable level intersections may be undersampled. This methodology thereby improves statistical robustness and ensures that identified biomarkers are not merely artifacts of underlying confounding variables. While only explicitly encoded covariates can be tested for, latent factors as well as interaction terms could also be incorporated by preprocessing the data to explicitly attach them, though doing so is left to the user at present. The package is optimized for high-performance computing through parallelization, allowing efficient processing of large datasets. Built-in visualization functionality of the package further enhances the immediate interpretability of results, providing a user-friendly implementation for assessing confounder-adjusted associations as well as to refine the variable pre-selection such as by clustering variables showing strongly similar co-association patterns.

We benchmarked metadeconfoundR on simulated metagenomic data, building on previous and extensive benchmarking efforts (Chen *et al.* 2023, Wirbel *et al.* 2024). The choice of metagenomic data for this purpose reflects the primary uses of metadeconfoundR in our own work, as well as how such data exhibits several challenging characteristics including compositionality, unequal sequencing depth bias, and frequent zero-inflation (Friedman and Alm 2012, Kurtz *et al.* 2015, Gloor *et al.* 2017, Hawinkel *et al.* 2019, Wirbel *et al.* 2024). The implementation is applicable for any type of numeric, binary data, or even ordinal variables by allowing for rank-based tests. Results on similar-sized datasets of immune cell frequencies, protein abundances or metabolite concentration appear qualitatively similar to those from metagenome abundances (Forslund *et al.* 2021, Maifeld *et al.* 2021), but caution should be exercised until these simulations have been performed. The previous benchmarks highlighted how several tools that in principle possess similar capabilities either do not control the false discovery rate well under these conditions or become very resource-intensive at typical cohort-scale biomarker discovery dataset sizes. Overall, more general methods, like linear models (and their expansions) or blocked Wilcoxon tests (as implemented in the coin package (Hothorn *et al.* 2008)), appear to be the better choice over those approaches trying to explicitly model specific data properties (Chen *et al.* 2023, Wirbel *et al.* 2024, Gamboa-Tuz *et al.* 2025, Pelto *et al.* 2025). We therefore principally compared metadeconfoundR to one leading covariate-adjustable differential abundance testing tool, namely MaAsLin2 (Mallick *et al.* 2021). While MaAsLin2 also uses linear mixed effects models to associate features to metadata variables, it differs from metadeconfoundR in that all potential covariates are included at once in a single multivariate model per feature. It also handles variable scaling differently, where metadeconfoundR defaults to rank transformations, MaAsLin2 defaults to log transformation with a pseudocount, with the option to alternatively model specific distributions like Zero-inflated Negative Binomial (ZINB) (Zhang *et al.* 2015). We also included a developmental version of the MaAsLin2 successor, MaAsLin3, but opted to focus on MaAsLin2, as the newer generation was not yet well documented when metadeconfondR was developed and was officially published just recently (Nickols *et al.* 2026).

As unequal sequencing depth bias is particularly relevant for metagenomic data, we ensured multiple mitigation strategies were compatible with metadeconfoundR’s design. One such strategy, rarefaction, has often been applied to equalize read counts across samples, but this approach discards valid reads from deeply sequenced samples, thereby reducing statistical power and potentially introducing new biases. While some studies argue that rarefaction ensures comparability (Schloss 2024), others regard the loss of information as highly problematic (McMurdie and Holmes 2014). Here, we add to this debate by using metadeconfoundR to assess the influence of unequal sequencing depth on differential abundance testing and the various normalization approaches’ ability to mitigate this influence.

MetadeconfoundR has already been extensively used in peer-reviewed publications, including large studies focusing on the gut microbiome (Forslund *et al.* 2021, Thirion *et al.* 2023b, Essex *et al.* 2024, Holle *et al.* 2025), metagenomes from low biomass settings (Kayongo *et al.* 2023, Cuthbertson *et al.* 2024), lipidomics (Jovanovic *et al.* 2024), flow cytometry (Dirks *et al.* 2026), multi-omics integration (Worm *et al.* 2022, Schoeler *et al.* 2023), and technical investigations (Bartolomaeus *et al.* 2021), demonstrating its robustness and broad applicability. The underlying design priority has been adoptability as standard practice, that is, to lower the barrier for as serious an attempt at adjusting for tracked potential covariates as possible without requiring either pre-specified hypotheses of confounding or prohibitively time-consuming manual inspection of each model or variable in turn. While this will not address every possible confounding influence, by acting as a conservative filter, the retained candidates typically constitute a much smaller set on which to conduct further tests, visualizations and interpretations in the light of specific domain knowledge.

## 2 Methods

### 2.1 Implementation

#### 2.1.1 Overview of the metadeconfoundR workflow

**Figure 1:**
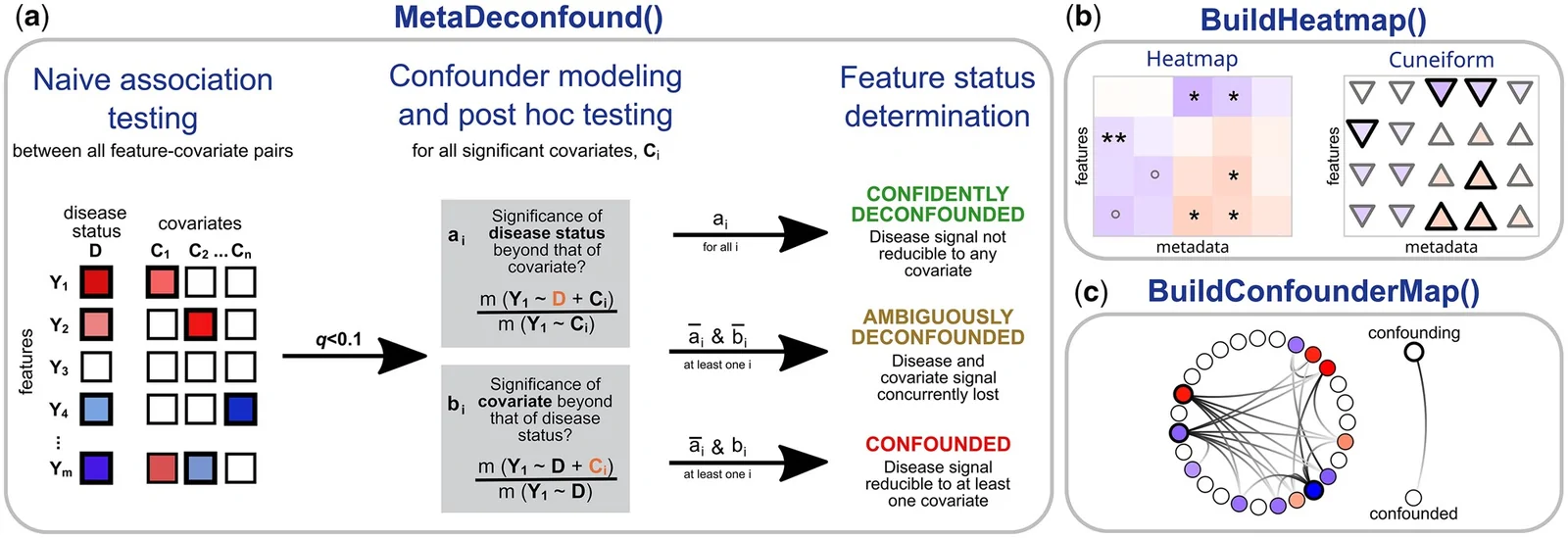
Workflow of the metadeconfoundR R package. (a) The function MetaDeconfound() performs the two-step statistical analysis: first by naive association testing between each feature and covariates and second the confounder modeling, post hoc testing and status labeling into i confidently deconfounded, ii ambiguously deconfounded or iii specifically confounded. (b) The function BuildHeatmap() can subsequently be used to summarize the output graphically. The heatmap represents each feature-metadata variable association in a single tile, indicating if this association is significant (*) or significant but confounded (°), and tile color indicating effect size and direction. (c) Circos plots visualizing confounder relationships between metadata variables associated with the same feature are generated with BuildConfounderMap(). Each circle represents a metadata variable with fill color indicating effect size and direction of the association with the respective feature. Thick circles highlight deconfounded associations. Gradient lines connect variables with a confounding-confounded relationship.

The metadeconfoundR workflow is designed to systematically identify and qualify associations between omics features and metadata variables in the presence of complex confounding structures. It comprises two main analytical steps: an initial screen for naive feature–metadata associations, followed by post-hoc model comparison to assess whether these associations can be reduced to confounding effects (Fig. 1a). The resulting classifications can be inspected and summarized using the package’s visualization functions (Fig. 1b and c), enabling iterative refinement of the input data representation and export of detailed results for downstream analysis. MetadeconfoundR is available as a well documented R package via CRAN or GitHub and requires merely two data frames as input containing the omics features and metadata variables.

#### 2.1.2 Initial “naive” association test and prior input

The first step of the metadeconfoundR analysis pipeline is the detection of naive (i.e. not explicitly accounting for covariates) associations between individual omics (dependent) variables (assumed numeric and continuous) and metadata (independent) variables (assumed binary/two-level nominal, categorical/multi-level nominal, ordinal or continuous). To increase robustness to non-normally distributed features, metadeconfoundR uses rank-based tests by default, reducing the need for any assumptions of a particular distribution. Specifically, the Mann-Whitney U test, Kruskal-Wallis test, or Spearman correlation test is applied when testing associations with two-level nominal, multi-level nominal, or (pseudo-)continuous variables, respectively. Users can explicitly define the data type of each metadata variable, but by default, metadeconfoundR automatically infers the data type using the following rules: Numeric variables containing only the values 0 and 1 (e.g. case/control status) are treated as two-level nominal; non-numeric variables (e.g. body site or country) are classified as multi-level nominal; and numeric variables with values beyond 0 and 1 (e.g. BMI or age groups) are treated as (pseudo-)continuous. Ordinal variables are treated as continuous variables under rank-transformation.

If the supplied features are binary (like presence-absence data) a likelihood ratio test is used instead to determine significance of naive associations: A generalized linear model with binomial error term containing only the respective metadata variable as independent variable is compared to a null model.

#### 2.1.3 Standardized effect size calculation

The effect size of each association is determined using Cliff’s Delta (Meissel and Yao 2024) for two-level nominal and Spearman’s rho for (pseudo-)continuous metadata variables. In case of binary features, Cliff’s Delta is used for (pseudo-)continuous and the Phi coefficient (Guilford 1941) for two-level nominal metadata variables. This design choice was made to ascertain a signed, standardized effect size defined on the same scale across variables, regardless of distribution. While there are some scale differences depending on whether a continuous vs binary association is calculated using the one or the other metric which the user should be mindful of, this is minor enough that relative comparison and visual inspection of effect sizes is straightforward with few edge cases to consider. Categorical variables, however, cannot be assessed in this manner and are flagged as such. The output of the first step is a data frame reporting raw *P*-values, multiple testing corrected *P*-values (using Benjamini-Hochberg adjustment by default), and non-marginal effect sizes for each association based on the naive tests (Fig. 1a, left).

Multiple testing correction, per default, is performed per metadata variable over the number of tested features with the option to change to a correction over the number of features and number of metadata variables.

#### 2.1.4 Post-hoc regression tests for independent effect of naive association drivers

**Figure 2:**
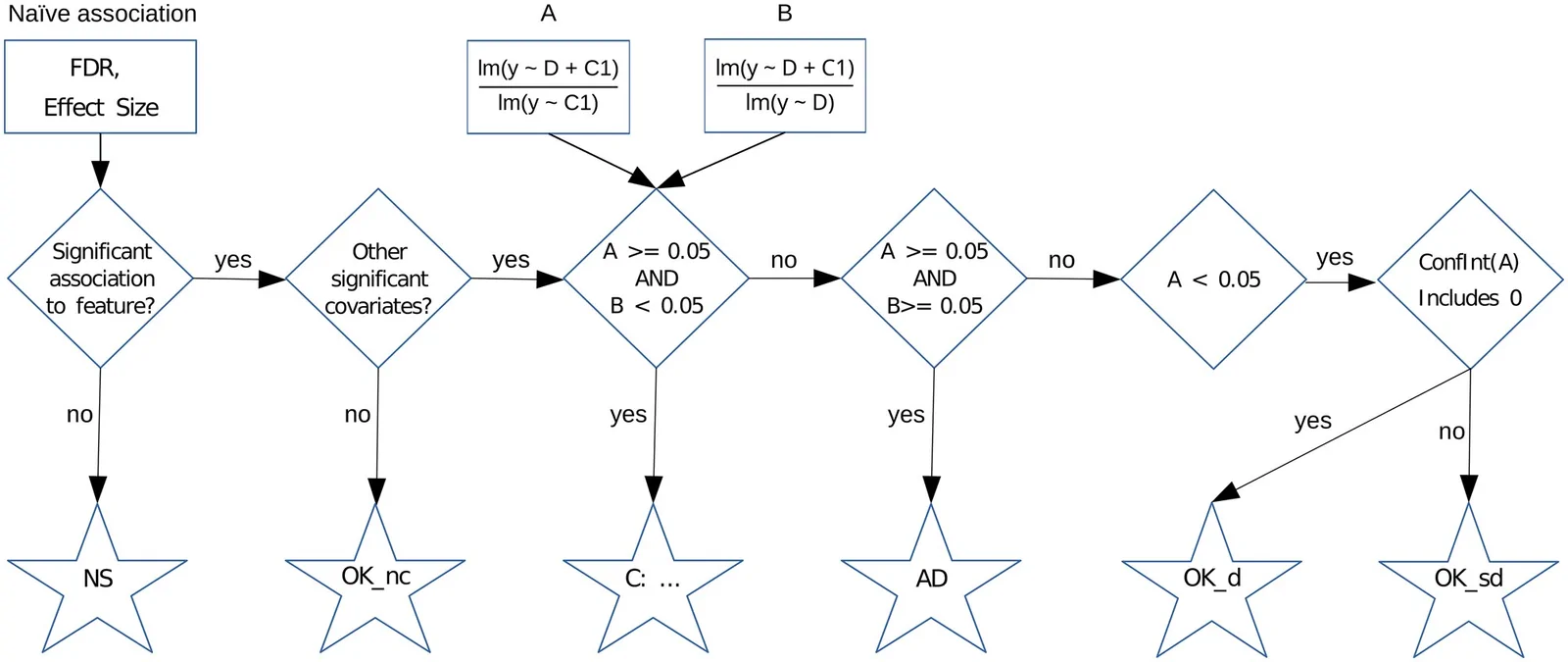
Association confounding status labeling decision tree. Based on the naive association results and the chosen significance and effect size cutoffs (top left) a label is assigned to each feature-metadata variable association. If other metadata variables are associated with the same feature, the confounder-confounding relationship between pairs of these metadata is determined using likelihood ratio tests (A and B). In case of random effects being supplied to the function call, parts marked in red are added to the decision tree.

In the second stage, metadeconfoundR identifies potential confounders through a systematic nested post-hoc model comparison approach. If only one independent variable is associated with a dependent variable, the former is considered “trivially deconfounded” with respect to the latter. Otherwise, for each inferred naive association between a feature (dependent variable, *Y* below) and a metadata variable (independent variable, D below) where one or more additional metadata variables ($C_{1}$-$C_{k}$, below) also shows such association, the association between D and Y is tested for potential confounding by any of $C_{1}$-$C_{k}$ (Fig. 2). Whether the association between $C_{i}$ and Y is considered depends on two user-defined thresholds: (1) a significance level (*P*-value, adjusted for multiple comparisons across the input feature space), and (2) a minimum absolute effect size.

For all resulting pairs ($C_{i}$ D) of metadata variables associated with Y, likelihood ratio tests (LRTs) of nested linear models for Y as a function of $C_{i}$ and/or D are conducted to assess whether the effect of D on Y could follow solely from D being a covariate of a more direct effect of $C_{i}$ on Y. The central assumption is that if variable $C_{i}$ is the true driver of a feature Y, with D merely a correlate of $C_{i}$, then $C_{i}$ should offer greater predictive power for Y than D does. Accordingly, three linear models are considered: a model including only $C_{i}$ (m_$C_{i}$: Y $∼C_{i}$), a model including only D (m_D: Y ∼ D), and a full model including both variables (m_full: Y $∼C_{i}$ + D).

The fits of these models are compared in two nested combinations (REML optimization is disabled so as to ensure LRTs remain correct in case a random factor is included):

- Reverse LRT: Compares m_$C_{i}$ vs m_full—does adding D improve prediction beyond $C_{i}$?
- Forward LRT: Compare m_D vs m_full—does adding $C_{i}$ improve predictive power over D alone?

To ensure flexibility across different study designs the user may specify a set of “universal” additional covariates included in every model, including random factors to account for dataset structure (shifting to a mixed-effects model), as well as additional parameters changing the error model e.g. to model Y using logistic regression. If random and/or fixed effects are supplied, metadeconfoundR performs an additional LRT to determine whether a naively significant association can be reduced to the random/fixed effects alone (Supplemental Fig. 1, available at supplementary material  *Bioinformatics Advances* online). If this is the case, the association is labeled as being not significant (“NS”) and nested confounder testing is skipped. If the association is not reducible to the random/fixed effects alone, nested confounder testing is performed, with the random/fixed effects included in all nested models.

If non-normalized count data (i.e. the sum of counts varies between samples) is supplied and the rawCount argument is set to TRUE, metadeconfoundR will use a tuple consisting of the feature Y and the sum of counts per sample as dependent variable in all built models. In this scenario, total sum scaling (TSS) is applied to the count data internally exclusively to perform naive association testing, as unadjusted counts cannot be used directly for naive tests and standardized effect size calculation due to unequal sequencing depth between samples. In all other scenarios, including the default behavior, no internal transformations are performed. A detailed overview of the expected input, performed transformations and employed methods for the different analysis modes can be found in Table 1. Further explanations and recommendations about input data preprocessing and choice of analysis mode are available at the package vignette: https://htmlpreview.github.io/?https://github.com/TillBirkner/metadeconfoundR/main/vignettes/Introduction\_To\_metadeconfoundR.html\#input-data-requirements.

**Table 1:** Comparison of tests and models used in different MetaDeconfound() modes.

Mode		Default	rawCounts	Logistic	clr_mode
Feature data type		Everything not like the other modes	Raw count data	Binary data (presence/absence)	CLR-like data*
Internal feature transformation for naive tests		None	TSS transformation	None	None
Continuous metadata	Test	spearman cor.test	spearman cor.test	glm(family = “binomial”) + lrtest()	pearson cor.test
	Effect size	Spearman’s Rho	Spearman’s Rho	Cliff’s Delta	Pearson’s r
Binary metadata	Test	wilcox.test	wilcox.test	glm(family = “binomial”) + lrtest()	t.test
	Effect size	Cliff’s Delta	Cliff’s Delta	Phi coefficient	Pearson’s r
Categorical metadata	Test	kruskal.test	kruskal.test	glm(family = “binomial”) + lrtest()	lm() + F-test
	Effect size	NA	NA	NA	NA
Rank transformation of features		Yes	No	No	No
Model type for LRTs	Without random effects	lm() + lrtest()	glm(family = “binomial”) + lrtest()	glm(family = “binomial”) + lrtest()	lm() + lrtest()
	with random effects	lmer() + lrtest()	glmer(family = “binomial”) + lrtest()	glmer(family = “binomial”) + lrtest()	lmer() + lrtest()

* “CLR-like” data are continuous quantitative data in which the distances between values carry meaningful information (e.g. CLR-transformed abundances). No ranking, transformation, or normalization is performed within metadeconfoundR in this mode. Therefore, users should supply data for which parametric linear models are considered appropriate.

As these LRTs are applied as a filter for whether the naive association (already adjusted for multiple hypothesis testing) between D and Y should be retained, their application constitutes post-hoc tests, so that the most conservative control of that initial test follows from less strict criteria applied to the LRTs. Accordingly, these LRTs are not adjusted for multiple tests, which additionally guarantees that controlling for more potential covariates never results in reduced effective power to conclude confounding or other reducibility to a covariate. This reflects a design priority of minimizing falsely attributing direct influence of D on Y.

Additionally, as LRTs are at risk in some cases of concluding spurious significance in case of sparse/overdispersed input data, metadeconfoundR excludes from consideration tests where tests for sparsity (for discrete variables) or low variance (for continuous variables) indicate this, according to user-specified thresholds (with defaults given as at least 5 samples need to have a value different from the variable’s median). Beyond these filters, to provide an additional warning where this may still take place, prompting further manual inspection where critical, confidence intervals are calculated for the slope of D in model m_D. If this interval contains zero, despite significance in the original naive test, a warning flag is considered and “OK_d” is assigned as the final label.

#### 2.1.5 Integrating pairwise regression tests to classify overall confounding status of associations

The results of all LRTs emerging from each independent variable D versus each of its dependent variables Y are then integrated as follows to yield a final status label to be used to filter out associations likely to reflect confounding influence or mediation by one or more other metadata variables.

If all reverse tests of D with respect to Y achieve significance, whether the corresponding forward tests do or do not, then D is considered “strictly deconfounded” with respect to Y. This should be interpreted as meaning that the effect of D on Y cannot be fully reduced to any other metadata variable included in the dataset, but those $C_{i}$ where the forward test achieved significance may independently affect Y such that some but not all of the apparent effect of D on Y may be attributable to them. Which variables $C_{i}$ have such effects after controlling for possible dependencies between them, in turn, is tested for and provided within the output as these series of post-hoc tests are looped, treating them also as the D variable throughout the full loop of MetadeconfoundR.

If instead at least one reverse test of D and $C_{i}$ with respect to Y fails to achieve significance, but with every corresponding forward test also failing to achieve significance, then the influence of D cannot be distinguished either way from any such $C_{i}$ from the available data using this method. This is expected in cases where covariates $C_{i}$ are so strongly linked to D that they functionally form a single entity, such as a disease with a single risk factor with perfect penetrance, a treatment initiated upon diagnosis without exception, or phenotype measurements which are separate operationalizations of the same (here nonrecorded) state. In this case, D is considered “ambiguously deconfounded” with respect to Y, with the intent that further conclusions must be drawn from more detailed domain knowledge. The recommended approach in this case is to inspect remaining co-association visually using the clustered heatmap metadeconfoundR functionality, inspecting such “blocks” of co-association as indicating possible redundancy of the metadata representation warranting a dimensionality reduction preprocessing step such as replacing strongly clustered parameters with a biologically feasible representative.

Finally, if for at least one $C_{i}$ the forward test achieves significance but the reverse test does not, this suggests that D’s apparent effect on Y is in fact explained by that $C_{i}$. In this case, D is flagged as confounded by $C_{i}$ with respect to Y (Fig. 1a, middle). As this test is performed over any $C_{i}$ initially also associated with Y, this means that if D is concluded confounded with respect to its effect on Y, one or more confounders $C_{i}$ will be listed as responsible for its apparent effect. Where several such $C_{i}$ are indicated, this may reflect several wholly independent drivers but more frequently either that one of them is the “true” confounder ($C_{0}$) of D on Y and the others in turn its covariates, or that each such $C_{i}$ are covariates of a latent (not encoded in the dataset) metadata variable which is the true effector. This is tested and reported as per the previous case described, with multiple reflections of a latent confounder thus flagged in itself as “ambiguously deconfounded” with respect to Y. Confounders that are themselves found to be confounded with respect to Y are removed from the list for the D ∼ Y association, ensuring that when the “true” confounder ($C_{0}$) is present in the dataset, its covariates will not be listed as additional confounders.

Thus, all combinations of metadata (independent) and feature (dependent) variables in the input dataset are assigned one of the labels described in Table 2. Additional descriptions on output interpretation are available at the package vignette: https://htmlpreview.github.io/?https://github.com/TillBirkner/metadeconfoundR/main/vignettes/Introduction\_To\_metadeconfoundR.html\#interpretation.

**Table 2:** Confounding status labels reported in the output of metadeconfoundR.

Label	Description
NA	Not possible to test due to missing or malformed data
NS	Not significant in the initial (naive) test; no associations
OK_nc	No other significant covariates; trivially deconfounded
C: [list]	Confounded/mediated by the listed metadata variables; examine corresponding output for each in turn to resolve possible redundancy
AD	Ambiguously deconfounded; cannot be distinguished from other metadata variable also ambiguously deconfounded with regards to this feature
OK_d	Strictly deconfounded; not reducible to any included covariate, but confidence interval testing raises risks of artifacts from sparsity/overdispersion
OK_sd	Strictly deconfounded; not reducible to any included covariate, regression model and initial test in agreement

#### 2.1.6 Extended output, visualization and integration options

Beyond the core association testing and confounder classification workflow, metadeconfoundR provides a set of optional functionalities supporting downstream interpretation and reuse of results. These include flexible output formats for intermediate and final results, optional reuse of intermediate association statistics, computation of partial effect size estimates to quantify unique variable contributions under collinearity, and graphical summaries of association and confounding structures (Fig. 1b and c). In addition, an optional omics integration mode enables joint analysis of multiple feature spaces. Detailed descriptions of these functionalities are provided in the Supplementary Methods, available at supplementary material  *Bioinformatics Advances* online.

#### 2.1.7 Benchmarking

A reliable evaluation of any confounder-aware association testing framework requires scenarios in which the ground truth is explicitly known. Only under such conditions is it possible to quantify error rates, compare performance against alternative tools, and understand how reliability shifts with changes in sample size, effect strength, number and strength of confounders, degree of multicollinearity of metadata, and data preprocessing choices. Because such conditions cannot be guaranteed in real datasets, simulation is required.

To ensure that simulated data were of high quality, we adapted previously peer-reviewed and published workflows rather than generating entirely new simulations. We first utilized a longitudinal dataset generated with SparseDOSSA2 (Ma *et al.* 2021) as published alongside the LongDat (Chen *et al.* 2023) tool. SparseDOSSA2 simulation is parametric, assuming zero-inflated log-normal distribution of microbial features, therefore making it potentially biased and less representative of real metagenomics data. Nonetheless, the generated dataset contains up to 16 covariates acting as potential confounders, making it representative of classical scenarios in clinical research with a multitude of confounding factors—a key setting for employing metadeconfoundR.

Additionally, we added a rudimentary metadeconfoundR implementation to the BAMBI (v0.99) (Benchmarking Analysis of MicroBiome Inference) benchmarking pipeline (Wirbel *et al.* 2024). This pipeline uses simulated data sets from the SIMBA (v0.5.4) (Simulation of Metagenomic data with Biological Accuracy) R package (Wirbel *et al.* 2024), which in turn uses non-parametric signal implantation through unbalanced resampling to add differential abundance signals into real-world data. The rudimentary metadeconfoundR implementation consists only of (a) naive Wilcoxon tests and (b) LRTs for linear mixed effects models including the potential confounder either as fixed or random effect. While this framework can only simulate a single confounding factor, it produces datasets more closely representing real world metagenomics data, allowing for less biased assessment of metadeconfoundR’s differential abundance testing core principle and comparison to other tools across a wide range of sample sizes and signal strengths. For further details see (Wirbel *et al.* 2024).

Previous studies have already addressed the comparisons between multiple tools for differential abundance (Chen *et al.* 2023, Wirbel *et al.* 2024, Pelto *et al.* 2025). We compared the performance of metadeconfoundR against tools and approaches based on linear mixed-effects models. Our primary comparison was against the package MaAsLin2 (v1.20.0).

#### 2.1.8 Simulation design

The longitudinal SparseDOSSA2 simulations were configured to represent cross-sectional case-control scenarios by reducing datasets to a single time point per subject. 100 iterations per combination of the following parameters had been simulated: strength of association between differentially abundant features and the case-control variable (Spearman’s rho: 0.2, 0.5), number of additional covariates (0, 1, 4, 16), and sample size (10, 20, 38, 75, 150, 300). In addition, different data preprocessing (none/raw, rawCount mode, rarefaction, total sum scaling (TSS), and centered log-ratio (CLR)) was applied. Each dataset consisted of 300 microbial features, of which 10% were simulated as differentially abundant. Strength of association between the case-control variable and each covariate was randomly selected to be 0.25, 0.5, 0.75 or 0.99. For further details see (Chen *et al.* 2023).

Additional versions were created for the TSS preprocessed simulations only. The strength of the association between the case-control variable and each covariate was randomly selected from the following ranges: (0.25, 0.5, 0.75) and (0.25, 0.5). This effectively removes highly collinear covariates from the data, making the scenarios more favorable to the multivariate approach of MaAsLin2.

To examine robustness against variation in metadata scaling, additional analyses introduced systematic shifts into covariate distributions after data transformation. This allowed probing whether metadeconfoundR remains stable when covariates differ in magnitude or distributional domain.

Unequal sequencing depth effects were assessed by including total read count per sample as an additional covariate in every run. The number of discovered associations between features and this sequencing-depth variable was counted overall and specifically among truly differentially abundant features. This allowed testing whether applied preprocessing methods (rarefaction, scaling, log-ratio transformations, etc.) mitigated depth-induced artifacts.

#### 2.1.9 Performance evaluation

For each simulated dataset, associations were tested with respect to a single predefined binary target variable D (e.g. disease or status), while all other metadata variables were treated as covariates and potential confounders. A true positive was defined as a feature correctly identified as associated with D when a direct effect was present in the simulation. If such an association was instead flagged as confounded by any of the covariates, it was instead counted as false negative. Features with less than five non-zero values were excluded, and associations with an adjusted *P*-value < 0.1 were defined as significant for all included tools. This anchoring to a single target variable D ensured that the benchmark reflects a confirmatory use case focused on identifying robust, non-confounded, disease-associated features rather than an all-versus-all exploratory search, in which case FDR correction would need to account not only for the number of tested features, but also the number of tested metadata variables.

Performance was summarized across repeated simulations using precision (TP/(TP + FP)) and recall (TP/(TP + FN)), with summary statistics computed as means across repetitions. For the BAMBI-based benchmarking, summary statistics were taken directly from the BAMBI pipeline output. Precision (1-FDR) rather than FDR was used to enable direct comparison with the benchmarking presented by (Wirbel *et al.* 2024).

For all SparseDossa2 simulations, detailed tool settings as well as evaluation methods can be found in the supplementary methods, available at supplementary material  *Bioinformatics Advances* online (Benchmarking: performance evaluation).

MetadeconfoundR and MaAsLin2 were both applied to analyze a public (Kayongo *et al.* 2023) dataset of 20,016S ampilcon sequencing samples of induced sputum from people living with HIV and/or COPD as a comparison of performance in real-world datasets containing highly collinear metadata. The curated set of metadata contained 9 variables including binary variables for gender, HIV and COPD status, and a combined double positive status (COPD AND HIV), as well as a continuous variable encoding duration of antiviral therapy (which all participants living with HIV received) and additional clinical variables.

## 3 Results

### 3.1. MetadeconfoundR confounder disentangling is on par with leading tools

To assess how metadeconfoundR performs in a realistic confounder-aware differential abundance setting, we integrated it into the established BAMBI benchmarking framework, which is based on implantation-based simulations with known ground truth. This benchmark specifically evaluates the ability of methods to identify true direct associations with a predefined target variable while avoiding false attribution of associations driven by confounding variables in a narrow confounding scenario representative of, e.g. a medication signal.

All simulations, parameter ranges, and tool configurations follow the BAMBI framework as previously described (Wirbel *et al.* 2024). In brief, BAMBI evaluates performance across repeated simulations while systematically varying key factors relevant to confounder disentangling, including the strength of association between the target variable and a confounder, sample size, and effect strength. Results reported here summarize metrics aggregated across simulation replicates, ensuring robustness and reducing the influence of stochastic variation.

**Figure 3:**
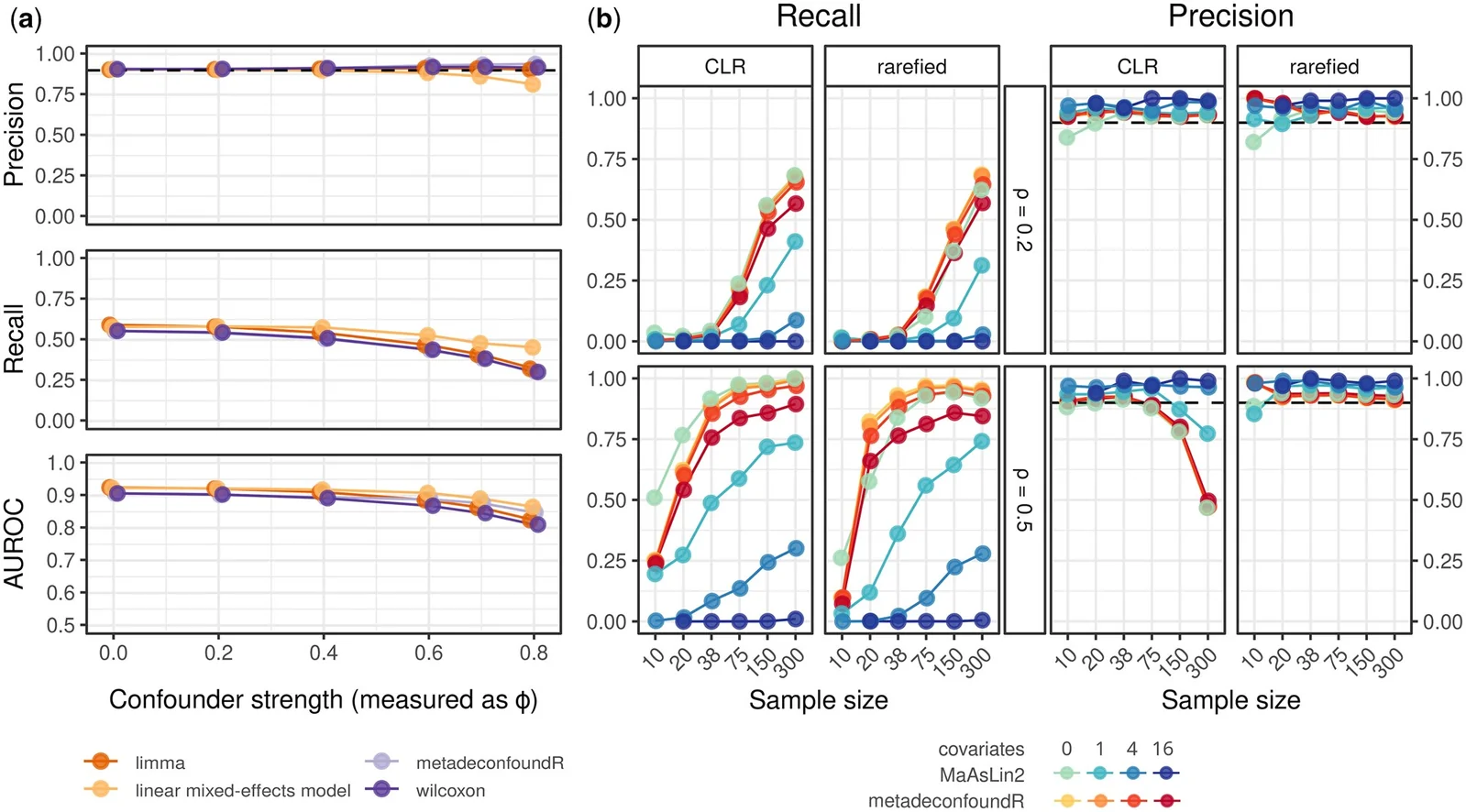
MetadeconfoundR remains accurate across covariate complexities. (a) Mean precision, observed recall, and area under receiver operating characteristic curve (AUROC) for univariate associations computed using metadeconfoundR, linear mixed-effect models, wilcoxon, and limma under 100 repetitions using a simulated data set (sample size = 200) generated using SIMBA and evaluated using BAMBI. (b) Mean recall and precision under different normalization strategies (Centered log-ratio (CLR) transformation or rarefying) for different sample sizes of count data simulated using sparseDOSSA2 under 100 repetitions. Individual panels show different preprocessing (left to right) and simulated strength levels of association (top and bottom).

Within this framework, metadeconfoundR was compared against methods previously identified as strong performers, including blocked Wilcoxon tests, linear mixed-effects models (LMMs, corresponding to MaAsLin2-style analyses), and limma (Fig. 3a). Performance was evaluated using precision, recall, and AUROC, defined relative to the known ground truth. In particular, calling a confounder-driven association a direct association with the target variable was counted as a false positive, while failing to detect a true direct association was counted as a false negative.

Across the tested range of confounder strengths, quantified by the phi coefficient between the target variable and the confounder (φ = 0–0.8), all methods maintained control of the false discovery rate at the nominal 10% level. Precision remained stable for Wilcoxon tests, limma, and metadeconfoundR, while LMM-based approaches showed a modest decrease in precision at higher confounder strengths (φ > 0.6). Recall and AUROC decreased with increasing confounder strength for all methods, reflecting the increasing difficulty of disentangling direct and indirect effects. At higher confounder strengths, LMMs exhibited slightly higher recall and AUROC compared to the other approaches.

Overall, these results indicate that, within the BAMBI benchmark setting, metadeconfoundR performs comparably to established methods in detecting true direct associations while avoiding confounder-driven false positives. This places metadeconfoundR on par with leading approaches in a well-characterized and previously validated benchmarking framework, providing a realistic baseline for its confounder disentangling performance.

### 3.2 MetadeconfoundR outperforms MaAsLin2 on multi-covariate cross-sectional data

To evaluate how confounder-aware methods behave as dataset complexity increases, we next assessed performance on cross-sectional simulations with multiple potential confounders. Using SparseDOSSA2-generated datasets, we benchmarked metadeconfoundR against MaAsLin2 in scenarios with up to 16 additional covariates, reflecting clinical study designs in which a primary status variable is accompanied by numerous correlated metadata variables (e.g. demographic, technical, or clinical factors). All results summarize repeated simulation runs and are evaluated relative to known ground truth.

In the absence of additional covariates, metadeconfoundR and MaAsLin2 showed comparable precision and recall across sample sizes, preprocessing strategies, and effect strengths, consistent with results from the BAMBI-based benchmarking (Fig. 3b, Supplementary Figs 2 and 3, available at supplementary material  *Bioinformatics Advances* online).

As the number of covariates increased, clear differences emerged between the methods. While metadeconfoundR maintained stable precision and recall across increasing covariate counts, performance of MaAsLin2 deteriorated substantially. In this setting, additional covariates represent variables correlated with the primary status variable but not directly driving feature abundance, requiring methods to correctly identify the true driver while avoiding false attribution to correlated covariates tested in parallel.

When assessing covariates correlated with the primary status variable (ρ = 0.75), independent associations are not expected, since the primary variable provides the more direct explanation of the simulated feature signal. Consequently, any associations classified as independently significant for the correlated covariate should be considered false positives. According to this definition, metadeconfoundR showed a false positive rate (FPR) that was approximately four percentage points higher than MaAsLin2 (Supplementary Fig. 5, available at supplementary material  *Bioinformatics Advances* online). However, the increase in FPR is an acceptable trade-off, as metadeconfoundR also provides additional insights (confounding assessment) rather than robust association detection alone, as performed by MaAsLin2. We additionally evaluated scenarios of additive confounding, where the combined explanatory contribution of two uncorrelated covariates exceeded that of either covariate individually. As expected, MaAsLin2 correctly attributed these associations to the confounding variables, whereas metadeconfoundR classified them as robust associations because it evaluates one potential confounder at a time. However, metadeconfoundR can indirectly detect these additive confounders by the partial effect sizes estimated with the GetPartialEffSizes() function. GetPartialEffSizes() correctly indicated that the simulated status variable contributed only a negligible amount of unique explained variance, thereby revealing the underlying additive confounding (Supplementary Fig. 6, available at supplementary material  *Bioinformatics Advances* online).

Across all tested conditions, very small sample sizes (*n* = 10) were insufficient to detect even strong associations (ρ = 0.5), while weaker associations (ρ = 0.2) were reliably identified only at sample sizes of 75 or greater. Both tools generally kept the precision above the predefined level of 0.9 (FDR < 0.1) except when processing CLR-transformed data with strong associations and large sample sizes. Here, precision dropped as low as 50 %.

Considering that MaAsLin2 implements a global FDR-adjustment instead of adjusting separately per metadata variable, and encounters difficulties with strongly correlated covariates, we tested how much of the performance lead of metadeconfoundR was attributable to these parameters. We, therefore, additionally benchmarked with maximal correlation of additional covariates C to the main signal variable D of 0.75 or 0.5 and summarized together with the results from the original simulation design (max. possible correlation 0.99) (Supplementary Fig. 4, available at supplementary material  *Bioinformatics Advances* online). MaAsLin2 performance was evaluated using the default/standard approach of global FDR correction (top half) and a per-metavariable FDR correction (bottom half). MaAsLin2 performance increased drastically with removal of strongly correlated covariates. Yet, recall levels of the single covariate (1_Maaslin2) runs remain below those of metadeconfoundR in the 16 covariate runs (16_metadeconofundR). While precision reaches the same or slightly higher levels than metadeconfoundR levels in datasets with at least 150 samples. Interestingly, switching to a per-metavariable FDR correction for MaAsLin2 leads to a subtler effect, slightly increasing recall rates while decreasing precision, closer matching the specified 0.9 precision value.

Finally, while metadeconfoundR and MaAsLin2 exhibited comparable baseline computational costs, metadeconfoundR benefited more strongly from parallelization, resulting in improved scalability to larger datasets (Supplementary Fig. 7, available at supplementary material  *Bioinformatics Advances* online).

### 3.3 Rarefaction is not inferior to other transformations

**Figure 4:**
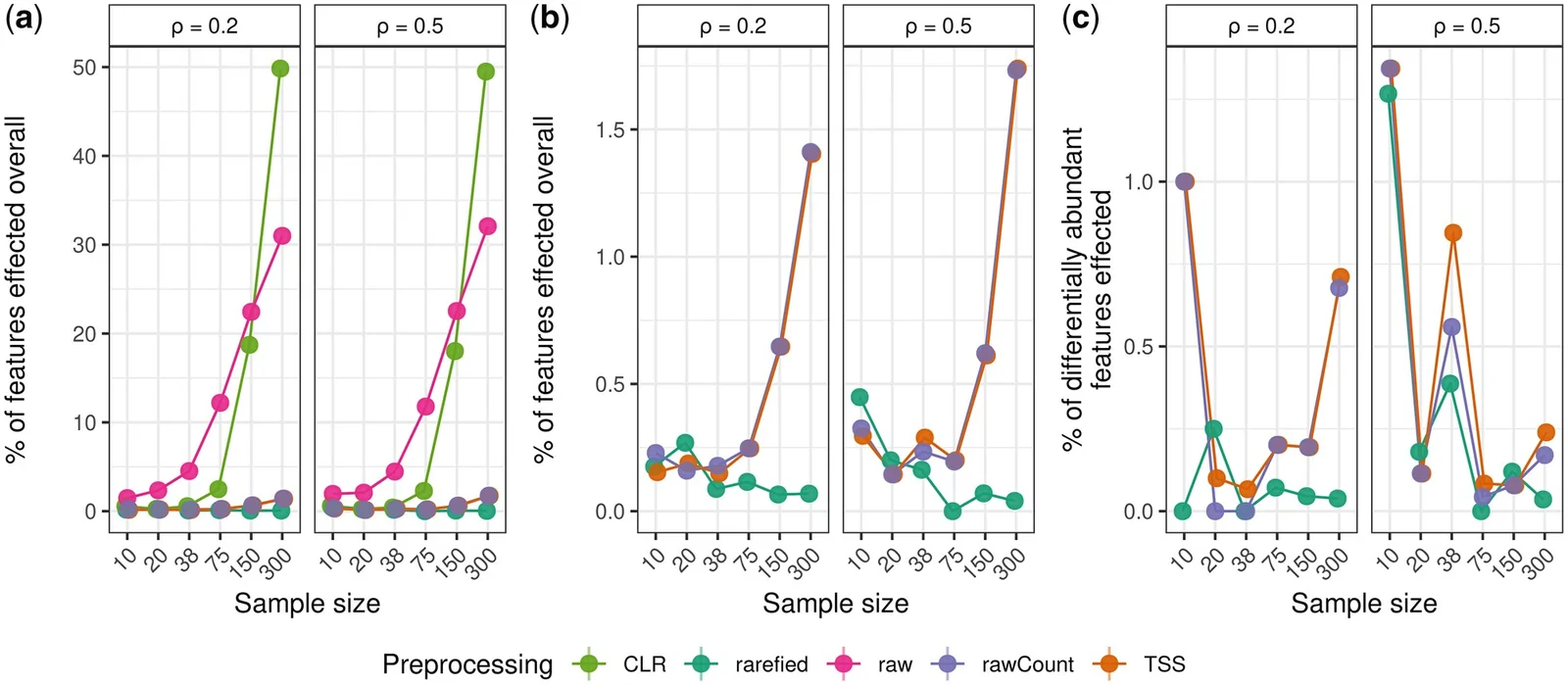
Rarefaction accounts well for unequal sequencing depth. (a) Proportion of features associated with sequencing depth out of all features for all preprocessing modes. (b) Zoomed in version of (a) with CLR and raw data removed. (c) Proportion of differentially abundant features associated with the sequencing depth out of all differentially abundant features. Based on metadeconfoundR analysis of SparseDOSSA2 simulated data including sequencing depth information as an additional covariate alongside the case-control variable and 16 simulated covariates.

Unequal sequencing depth remains a debated issue in metagenomic analyses, and various transformation strategies have been proposed as mitigation(McMurdie and Holmes 2014, Schloss 2024). We analyzed the extent to which different transformations reduced the impact of sequencing depth in our datasets. While raw count data exhibited a strong association with sequencing depth, rarefaction, total sum scaling (TSS), and metadeconfoundR’s rawCount mode were equally effective in minimizing these associations (Fig. 4, Supplementary Fig. 8, available at supplementary material  *Bioinformatics Advances* online).

CLR transformation (in combination with metadeconfoundR’s forced rank transformation) performed worse than raw data, as up to 40% of differentially abundant features were associated with sequencing depth, even though the CLR compatibility mode clr_mode was used (Fig. 4, Supplementary Fig. 8, available at supplementary material  *Bioinformatics Advances* online).

Even though rarefaction removes a substantial number of reads per sample (sometimes exceeding 99 % of a sample’s reads (Supplementary Fig. 9, available at supplementary material  *Bioinformatics Advances* online)), it did not negatively impact recall or precision in our analyses (Supplementary Figs. 2 and 3, available at supplementary material  *Bioinformatics Advances* online).

### 3.4 The metadeconfoundR R package offers robust and extensive functionality with high flexibility

Beyond its core functions, metadeconfoundR includes a set of complementary features designed to address practical challenges encountered in high-dimensional omics analyses. Partial effect size estimates allow quantifying the magnitude of direct associations after accounting for confounding, facilitating interpretation beyond statistical significance in the presence of (partial) collinearity. Summarizing heatmaps and circos plots provide compact visual representations of complex association patterns across features and covariates, supporting exploratory assessment of confounding structures and effect consistency. Extensive confidence interval testing ensures validity of confounder labelling results.

To accommodate diverse study designs and data types, metadeconfoundR further supports logistic regression for binary features like presence-absence data, a raw count mode tailored to microbiome data, and basic omics integration analyses for jointly assessing multiple feature spaces. These options, documented in detail in the accompanying vignette, address common scenarios in applied biomedical research, where outcome types, data preprocessing strategies, and omics layers vary substantially across studies.

In contrast to approaches that aim to explicitly model data distributions, metadeconfoundR prioritizes robustness across heterogeneous settings by applying a forced feature rank transformation followed by post-hoc nested model comparisons. Consistent with this design choice, the benchmarking analyses indicate that metadeconfoundR remains stable across heterogeneous preprocessing strategies and increasing covariate complexity, in contrast to fully multivariate model-based approaches.

### 3.5 Impact of metadata characteristics and sample size in metadeconfoundR performance

To evaluate the relative frequency of status labels assigned by metadeconfoundR, we summarized all labels assigned to associations between features simulated with differential abundant simulated features and the main signal variable. Across our simulations, between 5% and 80% of true associations were labeled AD, depending on the sample size, the association strength and the number of included metadata variables. For a high level of association between covariates (ρ=0.99), we observed the highest proportion of ambiguously deconfounded associations. As the association between metadata variables decreased (ρ < 0.75), the proportion of strictly deconfounded features increased (Supplementary Fig. 10, available at supplementary material  *Bioinformatics Advances* online).

We additionally evaluated how different effect size thresholds influence metadeconfoundR performance by re-analyzing a subset of our simulations, considering associations with effect sizes below 0.1 or 0.3 as non-significant. At an effect size threshold of 0.3, false positives could be substantially reduced and were completely eliminated at higher sample sizes. The improvements in precision are at the cost of reducing recall rates. However, the decrease in recall was less pronounced than the corresponding improvement in precision (Supplementary Fig. 11, available at supplementary material  *Bioinformatics Advances* online).

As an example of performance differences between metadeconfoundR and MaAsLin2 when analyzing real-world data containing highly collinear metadata, we applied both tools to a published 16S amplicon sequencing HIV/COPD dataset. While metadeconfoundR identified a wide range of robust, as well as a large portion of confounded or ambiguously deconfounded associations with two of the included metadata variables being strongly collinear, MaAsLin2 reported no significant association. Only after manually performing per-metadata multiple testing correction of raw *P*-values reported by MaAsLin2, a low number of associations remained significant, only partly matching metadeconfoundR results (Supplementary Fig. 12, available at supplementary material  *Bioinformatics Advances* online).

Combining, these results provide users with further guidance on expected performance and how to interpret the output when applying metadeconfoundR.

## 4 Discussion

MetadeconfoundR offers a statistically rigorous and computationally efficient solution for confounder-aware biomarker discovery. Our tool is intended as an exploratory covariate analysis framework that facilitates systematic screening for potentially confounded associations across large numbers of features and metadata variables. Its performance in settings with few covariates is comparable to leading microbiome confounder-aware differential abundance methods, such as blocked Wilcoxon tests and linear models, while offering additional robustness and flexibility in a user-friendly implementation. MetadeconfoundR has as intended user group researchers performing exploratory analyses of high-dimensional case-control datasets, particularly in omics applications where large numbers of molecular features must be evaluated against clinical metadata. Compared to other established linear model-based approaches like MaAsLin2, metadeconfoundR performs significantly better as the number of covariates increases. This sustained performance in multi-covariate settings is enabled by metadeconfoundR’s unique approach of fitting individual, small models per covariate pair instead of a single multi-covariate model, thereby minimizing the risk of model fitting failure due to, for example, multicollinearity. Additionally, the initial screening for naive feature-covariate associations helps reduce the overall computational load. This highlights a key advantage of metadeconfoundR as our approach reduces the need for potentially bias-inducing preprocessing steps like covariate selection. The tool remains stable without such preprocessing while neatly visualizing collinearity/redundancy structures in the data.

MetadeconfoundR combines the robustness of non-parametric approaches like the Wilcoxon test with the flexibility of (generalized) linear (mixed effects) models by rank-transforming all features. This eliminates further sources of bias, as less assumptions about the data are being made, and no preprocessing procedures to account for non-normality, zero-inflation, overdispersion or differently scaled covariates are needed. In addition, it enables the application of metadeconfoundR to a wide range of omics data and covariates, instead of being adapted to a specific data type and its *assumed* properties.

The generalist approach of metadeconfoundR positions our tool as a means to screen various omics spaces for confounding effects and gain insights into data structures without requiring in-depth domain expertise. However, we acknowledge that other approaches, each tailored to a specific omics space and its properties, may be more effective in their respective domains. Omics-specific preprocessing and transformation procedures should be performed prior to applying metadeconfoundR, using approaches that are appropriate for the data type in question. It is also important to note that, while several omics analysis frameworks employ multivariate approaches that jointly model dependencies across features and covariates (Argelaguet *et al.* 2018, Singh *et al.* 2019, Mallick *et al.* 2021), they are designed for different purposes. MetadeconfoundR is designed to detect monotonic associations between individual features and metadata variables, and is therefore primarily intended as an interpretable screening and hypothesis-generation tool. One major advantage of this approach is its robustness towards incomplete or missing data, reducing the need for potentially bias-inducing data imputation or complete removal of parts of the dataset. As a drawback, more complex, non-monotonic, or multivariate associations may go undetected. For example, metadeconfoundR does not explicitly model additive confounding, where the combined explanatory contribution of multiple covariates exceeds that of any individual covariate. In such cases, a confounded association may initially be misclassified as robust because confounding is assessed for one covariate at a time. To facilitate an indirect identification of these scenarios the metadeconfoundR package includes the function GetPartialEffSizes() that quantifies the variance uniquely attributable to each metadata variable. Associations driven primarily by additive confounding are therefore expected to show markedly reduced partial effect sizes relative to the initial effect estimates, allowing such cases to be identified during downstream interpretation. In addition, the metadeconfoundR framework can also be applied to derived variables, such as principal coordinates, combined metadata variables, or other dimensionality-reduced representations, enabling exploratory analysis of multivariate data summaries.

A challenge unique to metagenomic count data, that is not addressed by metadeconfoundR’s rank transformation, is the unequal sequencing depth across samples. Despite its prevalence, the best method to account for this imbalance computationally remains under debate. One widely used approach, rarefaction, has sparked considerable controversy, with opinions ranging from labeling it “inadmissible” (McMurdie and Holmes 2014) to advocating it as “best practice” (Schloss 2024). To contribute additional evidence to this ongoing discussion, we systematically compared common normalization and transformation strategies. We found that rarefying performs comparably to alternatives such as TSS, both in terms of their ability to mitigate effects of unequal sequencing depth, and their influence on the general performance of metadeconfoundR. Interestingly, CLR transformation exacerbated the influence of unequal sequencing depth. While CLR is known to artificially inflate low abundant taxa (Gamboa-Tuz *et al.* 2025), this seems potentiated in combination with the subsequent rank transformation of data, rendering CLR not compatible with metadeconfoundR default behavior. Introducing an alternative parametric metadeconfoundR mode alleviates this underperformance, making the tool compatible with preprocessing approaches that intentionally encode biologically relevant information in quantitative distances between observations, such as CLR transformation.

In summary, metadeconfoundR provides a robust, scalable, and interpretable framework for confounder-aware biomarker discovery in complex, high-dimensional omics datasets. Its stability in multi-covariate settings, low preprocessing demands, and compatibility with diverse study designs make it a valuable addition to the current landscape of differential abundance analysis tools. By reducing the risk of confounding and offering user-friendly visualization and interpretability features, metadeconfoundR empowers researchers to derive more reliable biological insights from their data. The substantial number of published studies across multiple omics domains that have cited and used metadeconfoundR show that the intended user group has adopted the tool for their analyses (Bartolomaeus *et al.* 2021, Forslund *et al.* 2021, Clos-Garcia *et al.* 2022, Eckenberger *et al.* 2022, Fromentin *et al.* 2022, Worm *et al.* 2022, Fan *et al.* 2023, Hua *et al.* 2023, Kayongo *et al.* 2023, 2024, Li *et al.* 2023, Robinson *et al.* 2023, Samuelsson *et al.* 2023, Schoeler *et al.* 2023, Thirion *et al.* 2023a, 2023b, Wu *et al.* 2023, Cuthbertson *et al.* 2024, Essex *et al.* 2024, Jovanovic *et al.* 2024, Kulecka *et al.* 2024, 2025, Van Rossum *et al.* 2024, Adriouch *et al.* 2025, Franz *et al.* 2025, Giner-Pérez *et al.* 2025b, 2025a, Grąźlewski *et al.* 2025, Holle *et al.* 2025, Knoll *et al.* 2025, Kosińska-Kaczyńska *et al.* 2025, Olweny *et al.* 2025, Russo *et al.* 2025, Versnjak *et al.* 2025, Wang *et al.* 2025, Dirks *et al.* 2026), underscoring its practical relevance across a range of biomedical research contexts.

## Supplementary Material

vbag242_Supplementary_Data

## Data Availability

All input data used in this work was already published by (Chen *et al.* 2023) and (Wirbel *et al.* 2024) for the SparseDOSSA2 and SIMBA/BAMBI simulations, respectively. Aggregated results are archived at Zenodo: https://doi.org/10.5281/zenodo.18470337.

## Code availability

The metadeconfoundR package (v.1.0.5) is available on GitHub: https://github.com/TillBirkner/metadeconfoundR and CRAN: https://cran.r-project.org/package=metadeconfoundR, with more in-depth tutorials and explanations of input options also available at these sites. The scripts used to perform all analyses and visualizations of this study, alongside a renv.lock file to reproduce the R environment, are available on GitHub: https://github.com/TillBirkner/metadeconfoundR_benchmarking. In addition, we provide a fully reproducible Docker image of our analysis environment, available on Docker Hub: https://hub.docker.com/repository/docker/tillbirkner/metadeconf-bench-env/general and archived at Zenodo: https://doi.org/10.5281/zenodo.18470337.
