# Supplementary material for: metadeconfoundR: Covariate analysis of high-dimensional cross-sectional omics data
Source: Bioinform Adv. 2026 Aug 19;6(1):vbag242. doi: 10.1093/bioadv/vbag242 (PMC13581263; doi:10.1093/bioadv/vbag242)
Supplement: Supplementary file 1 [file vbag242_supplementary_data.pdf]

## metadeconfoundR: covariate analysis of high-dimensional cross-sectional omics data

Till Birkner[1,2,3,4], Chia-Yu Chen[1,2,3], Morgan Essex[1,2,3], Kilian Dahm[5,6], Ulrike Löber[1,2,3,4], Thomas Ulas[5,6,7], Víctor Hugo Jarquín-Díaz[1,2,3,*], Sofia Kirke Forslund-Startceva[1,2,3,4,8,*]

1. Max-Delbrück Center for Molecular Medicine in the Helmholtz Association (MDC), Berlin, Germany
2. Experimental and Clinical Research Center (ECRC), a cooperation of the Max-Delbrück Center and Charité–Universitätsmedizin, Berlin, Germany
3. Charité–Universitätsmedizin Berlin, a corporate member of Freie Universität Berlin and Humboldt-Universität zu Berlin, Berlin, Germany
4. German Center for Cardiovascular Research (DZHK), partner site Berlin
5. Systems Medicine, German Center for Neurodegenerative Diseases (DZNE), Bonn, Germany
6. Genomics and Immunoregulation, Life & Medical Sciences (LIMES) Institute, University of Bonn, Germany
7. Platform for Single Cell Genomics and Epigenomics at the German Center for Neurodegenerative Diseases (DZNE), the University of Bonn and Western German Genome Center (WGGC), Bonn, Germany
8. Structural and Computational Biology Unit (SCB), EMBL Heidelberg, Germany

* Corresponding author(s): Sofia Kirke Forslund-Startceva & Víctor Hugo Jarquín-Díaz. Max-Delbrück Center for Molecular Medicine (MDC), Experimental and Clinical Research Center (ECRC) Charité, Campus Buch. Lindenberger Weg 80, 13125, Berlin, Germany.

**E-mail(s):** Sofia.Forslund@mdc-berlin.de; VictorHugo.JarquinDiaz@mdc-berlin.de

### Supplementary Material

- **Supplementary Figures**
  - Suppl. Fig 1- 12

- **Supplementary Methods**
  - Implementation: output of intermediate and final results
  - Implementation: visualization of results for inspection and refinement of analysis
  - Implementation: omics integration mode
  - Implementation: parametric analysis
  - Benchmarking: Simulation design
  - Benchmarking: performance evaluation

## Supplementary Figures

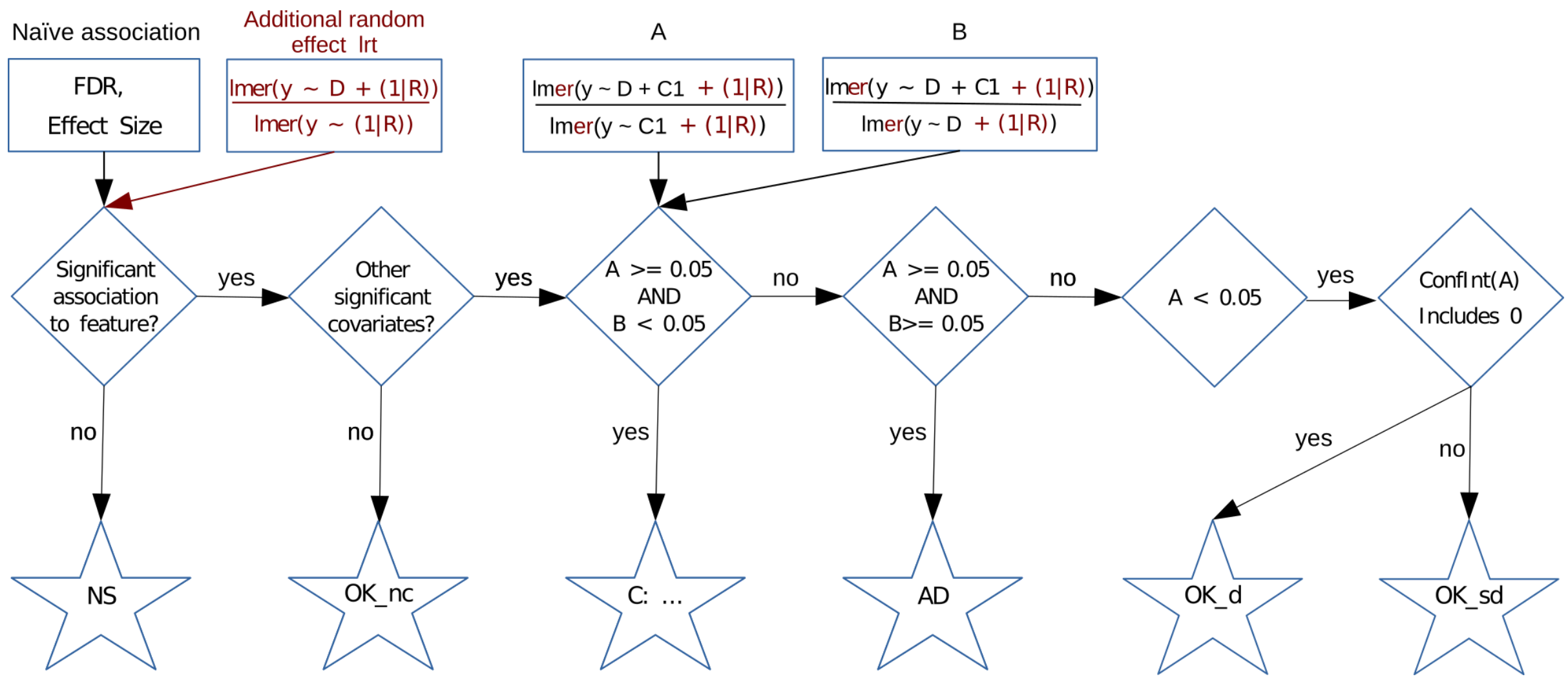


**Suppl. Figure 1: Association confounding status labeling decision tree including random effect elements.** Based on the naive association results and the chosen significance and effect size cutoffs (top left) a label is assigned to each feature-metadata variable association. If other metadata variables are associated with the same feature, the confounder-confounding relationship between pairs of these metadata is determined using likelihood ratio tests (A and B). In case of random effects being supplied to the function call, parts marked in red are added to the decision tree.

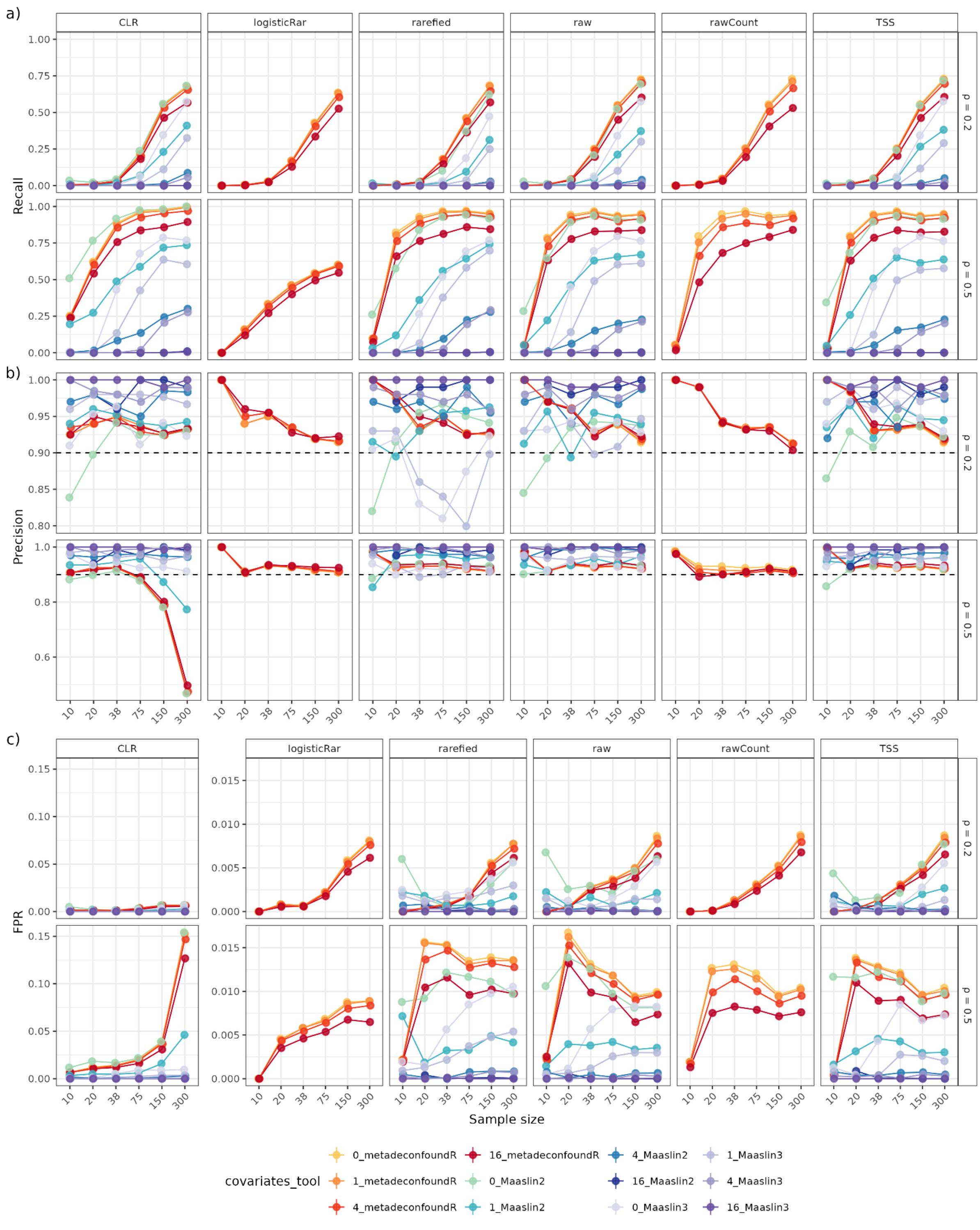


**Suppl. Figure 2: Full benchmarking performance of metadeconfoundR, MaAsLin2, and MaAsLin3 on sparseDOSSA2 simulated data.** Individual panels show different preprocessing (left to right) and association strengths (top and bottom). **a)** Recall **b)** Precision **c)** false positive rate (FPR). Each data point is the mean of 100 repetitions.

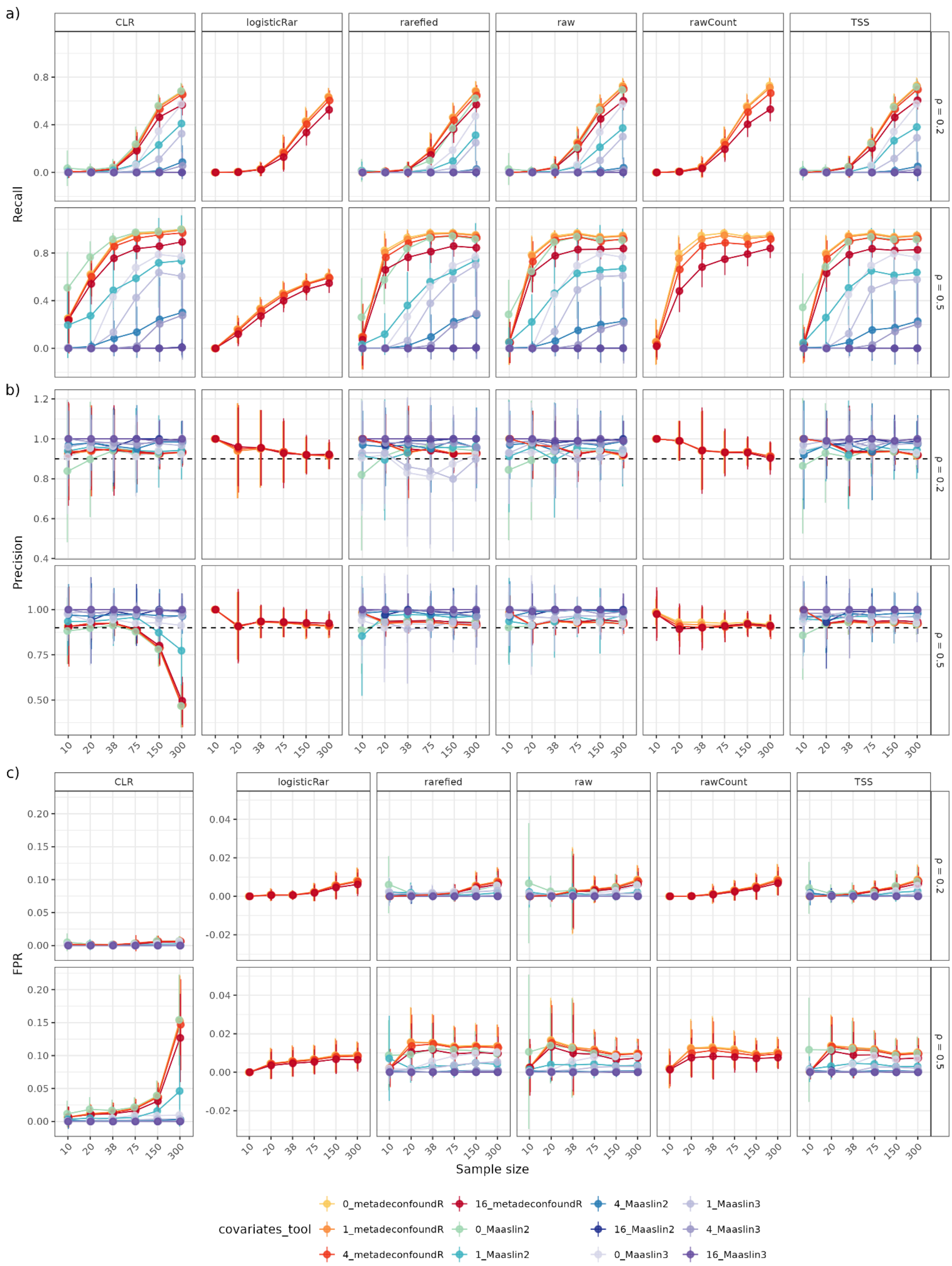


**Suppl. Figure 3: Full benchmarking performance of metadeconfoundR, MaAsLin2, and MaAsLin3 on sparseDOSSA2 simulated data with standard deviation as error bar.** Individual panels show different preprocessing (left to right) and association strengths (top and bottom). **a)** Recall **b)** Precision **c)** false positive rate (FPR). Each data point is the mean of 100 repetitions with error bars showing ± one standard deviation.

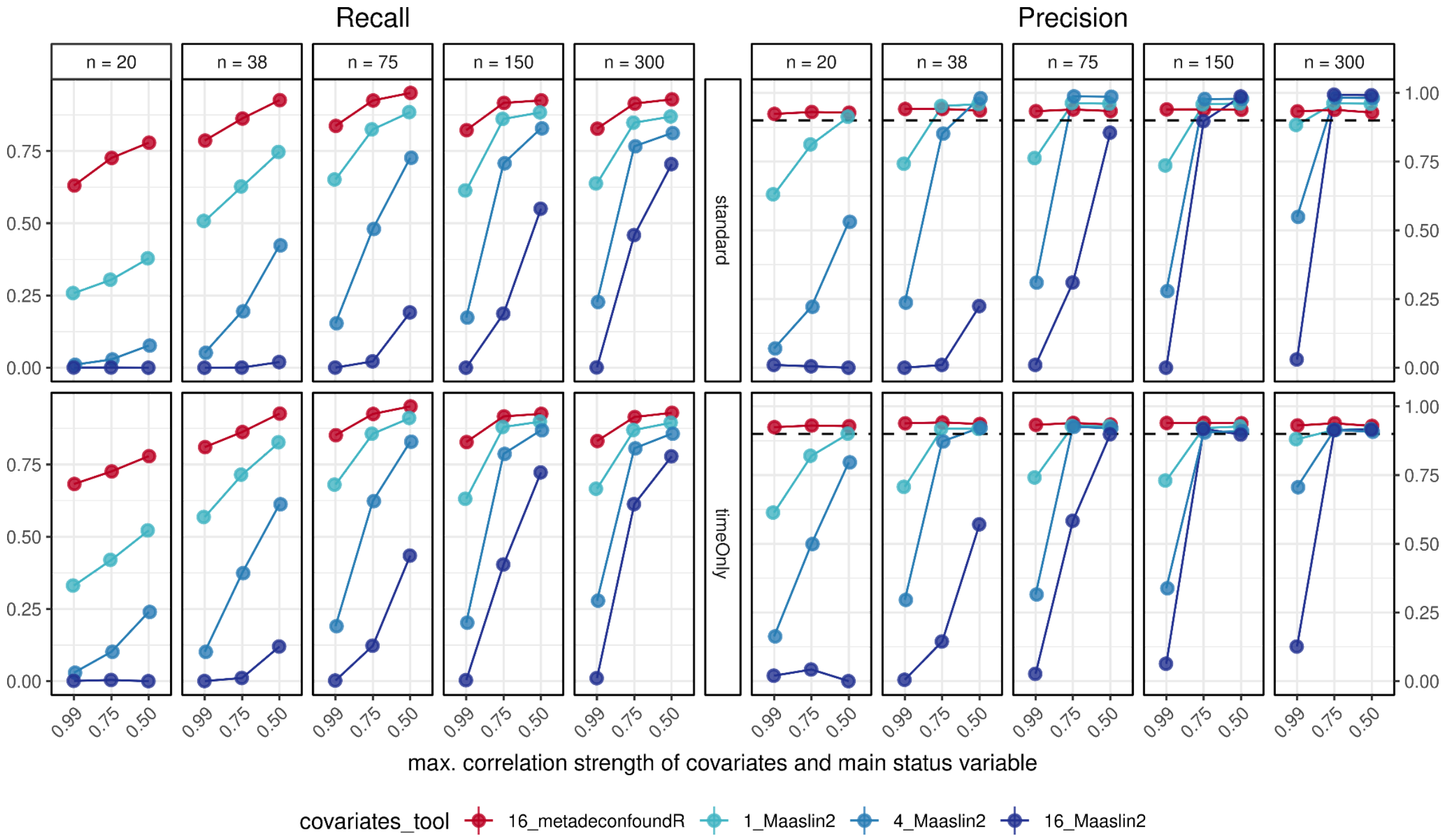


**Suppl. Figure 4: Maaslin2 performance strongly depends on confounder strength and FDR correction approach.** Recall (left panel) and precision (right panel) for TSSnormalized simulated datasets with main signal association strength of Spearman's rho = 0.5, and 1,4, or 16 additional covariates. Correlation strength between main signal variable and individual covariates was randomly selected to be rho = {0.25, 0.5, 0.75, 0.99}, {0.25, 0.5, 0.75}, {0.25, 0.5} for the left, middle, and right side of each sub-plot. Sample size of the simulated datasets is faceted along the x-axis direction in each panel. The upper half shows data based on the default FDR correction method of MaAsLin2 (correct for overall number of tests: n(features) x n(metadata variables)), while lower half shows data based on FDR correction for the number of features.

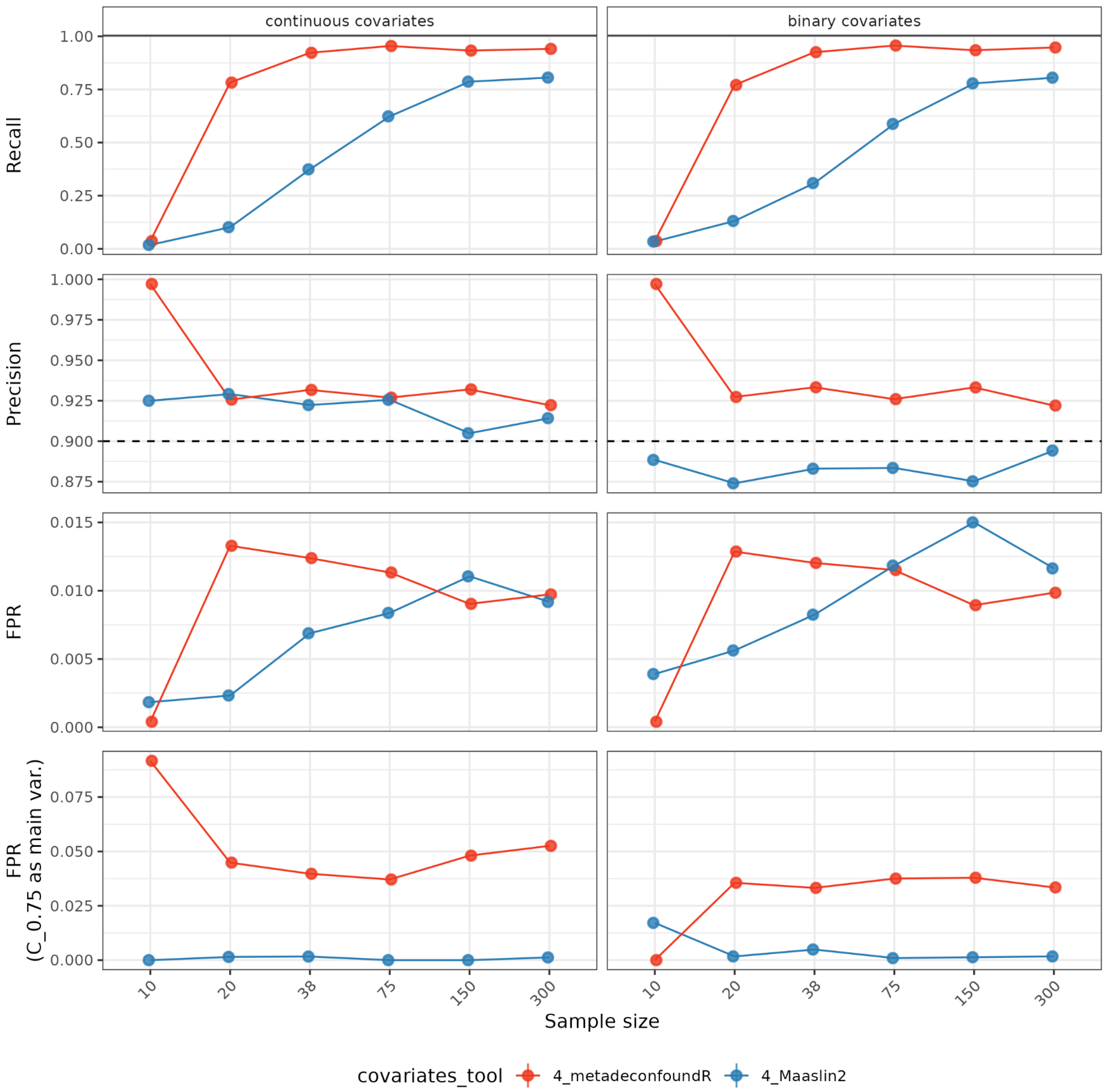


**Suppl. Figure 5: MaAsLin2 and metadeconfoundR performance differs slightly for continuous vs. binary covariates.** Subset of simulations to TSS normalized datasets with main signal association strength of Spearman's rho = 0.5, and four additional covariates. The bottom row shows false positive rate (FPR) when status of one of the covariates is determined instead of the main signal variable D (i.e. all associations should be labeled as non-significant or confounded by D, and all other significant associations are therefore false positives). The panels on the left side represent the standard set-up of all other datasets simulated using sparseDOSSA2. Alongside the binary main status variable, continuous covariates are simulated with specified correlation to the main status variable. The panels on the right side represent an alternative switched setup that simulates binary covariates. This setup was necessary because simulating continuous variables from a binary variable introduces additional variation. As shown in the bottom row, switching from continuous to binary covariates reduces the difference in FPR between the two tools in the "switched" scenario, while minimally affecting the metrics of the main signal variable D (first three rows).

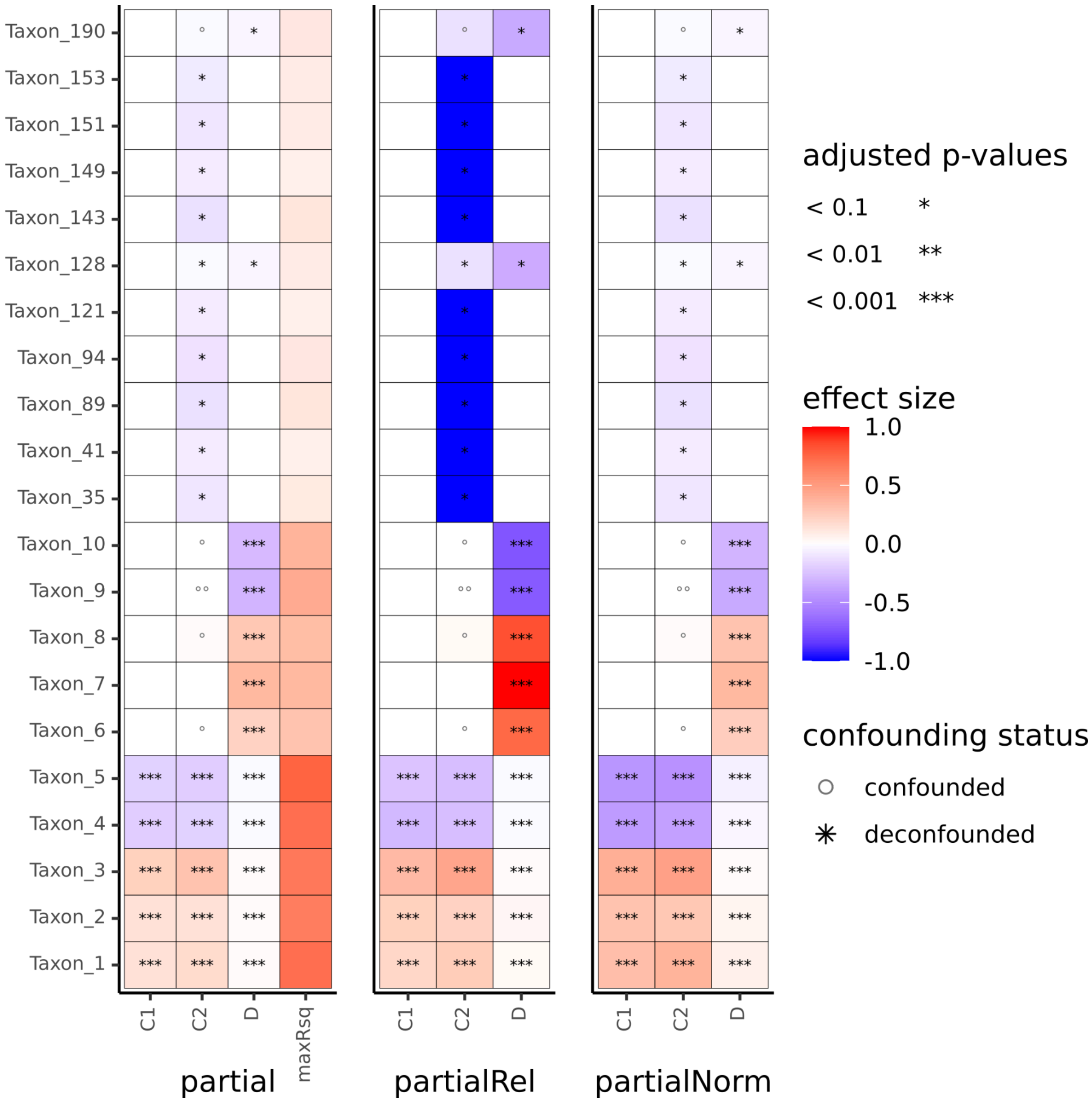


**Suppl. Figure 6: Indirect detection of additive confounding by metadeconfoundR.** Heatmaps generated by BuildHeatmap() function based on output of GetPartialEfSizes(). Significance based on tests performed within MetaDeconfound() function. Effectsize (tile fill color), here, indicates $R^2$ values (left) or proportions of $R^2$ values (center, right) with absolute range of 0-1 and sign indicating association direction. MaxRsq column in the left heatmap indicates $R^2$ values for the full model, i.e., the highest level of explained variance of a feature when including all univariately associated metadata variables as predictors. Although initial MetaDeconfound() run misclassifies associations of taxa one to five with D as strictly deconfounded, partial effect size uniquely attributable to D is minimal, indicating additive confounding effects within the set of included metadata.

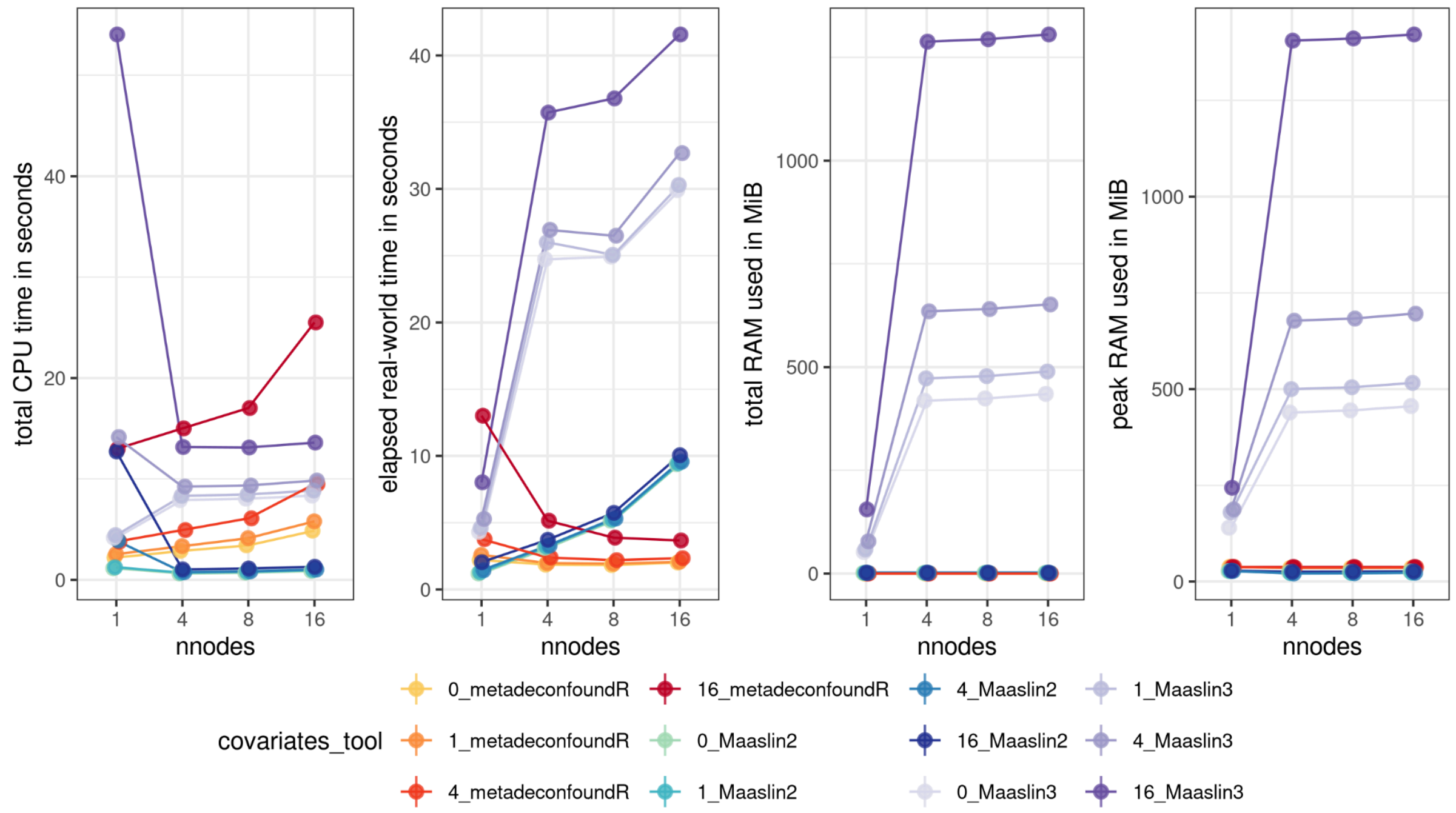


**Suppl. Figure 7: Resource needs for analyzing one 300 sample simulated dataset with 0-16 covariates using 1-16 CPUs (nnodes) for parallel processing.** Each data point is the mean of 100 repetitions.

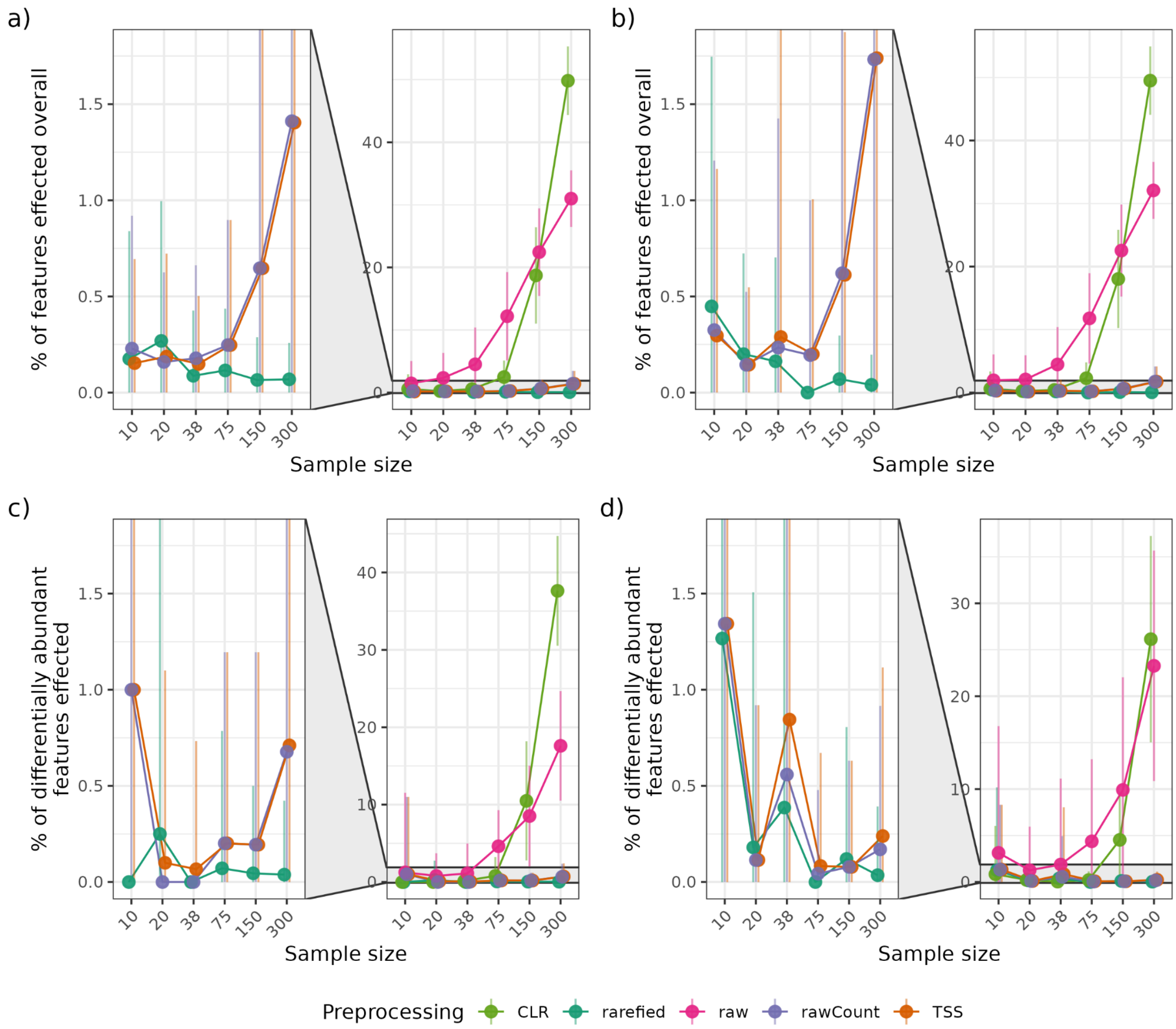


**Suppl. Figure 8: retained unequal sequencing depth bias under different preprocessing approaches.** Each subfigure shows complete data on the right side and a zoomed in version on the left side. Based on metadeconfoundR analysis of SparseDOSSA2 simulated data including sequencing depth information as an additional covariate alongside the case-control variable and 16 simulated covariates. Each data point is the mean of 100 repetitions with error bars showing ± one standard deviation. **a)** Proportion of features associated with sequencing depth out of all features for ground truth association strength ρ = 0.2 and **b)** for ρ = 0.5. **c)** Proportion of differentially abundant features associated with the sequencing depth out of all differentially abundant features for ground truth association strength ρ = 0.2 and **d)** for ρ = 0.5.

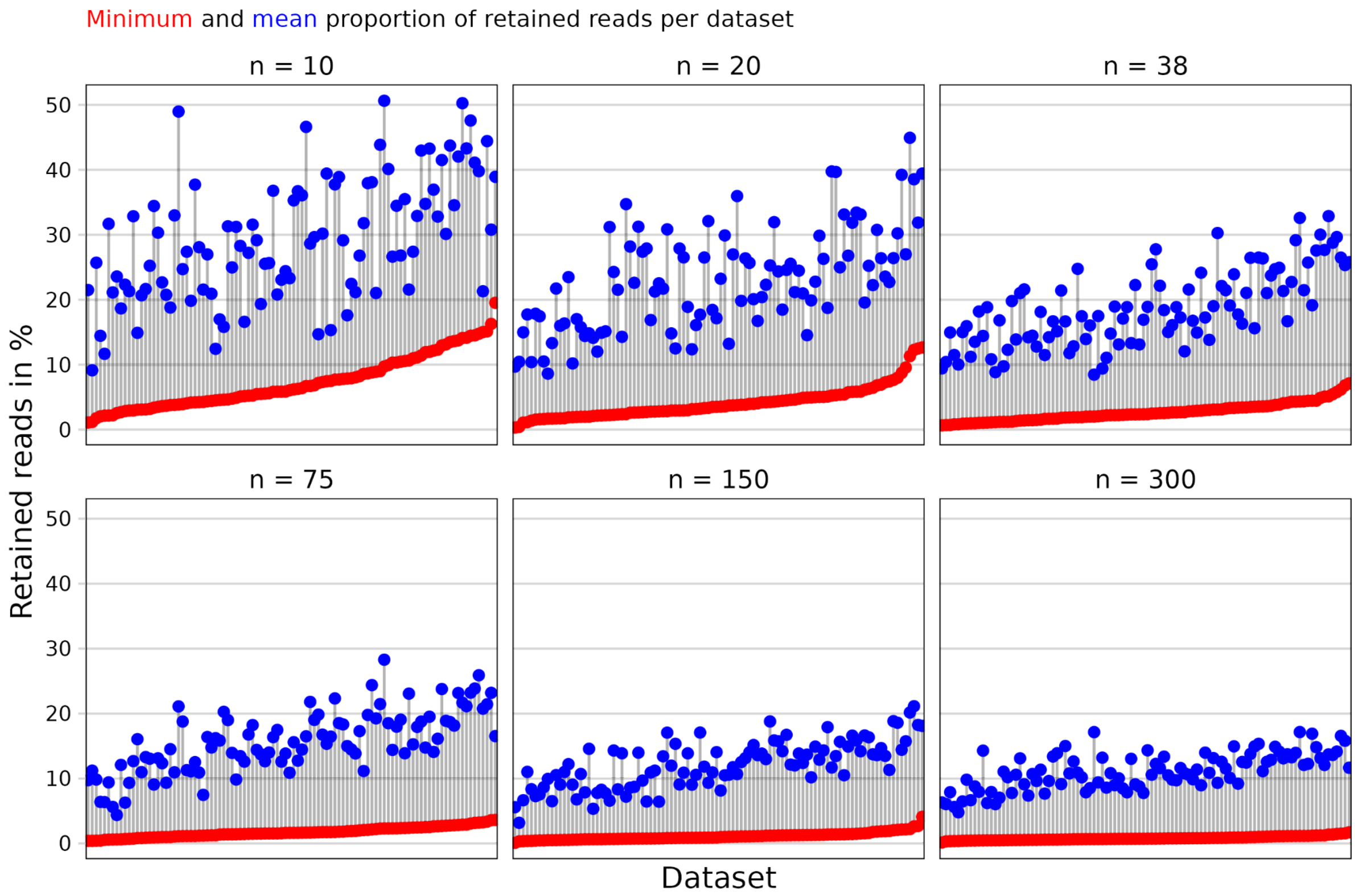


**Suppl. Figure 9: Rarefaction, on average, removes up to 90 % of reads in the simulated datasets created with sparseDOSSA2.** Individual panels each show 100 simulated datasets with the respective sample size n.

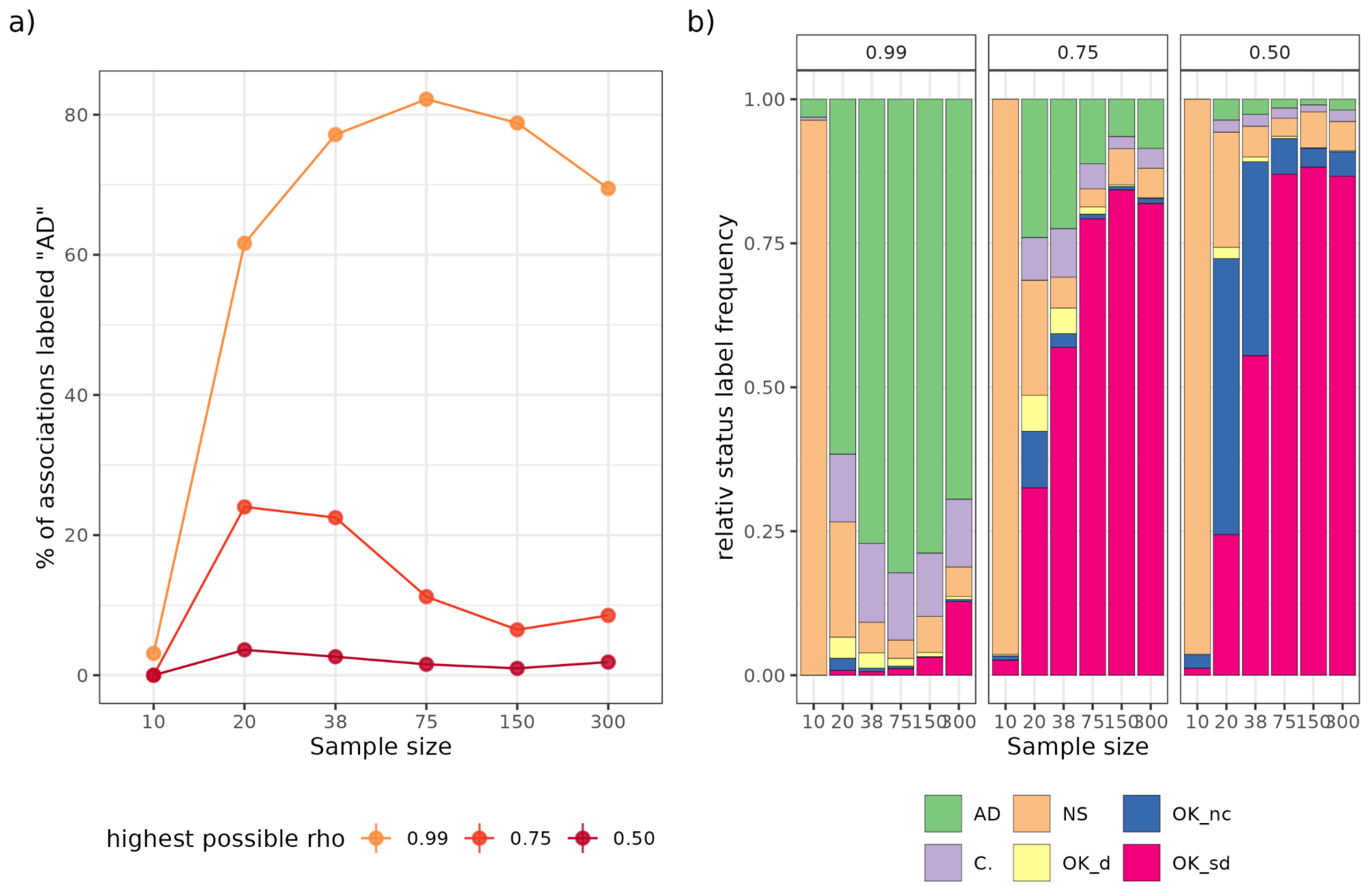


**Suppl. Figure 10: The prevalence of the different status labels strongly depends on the included metadata.** Relative frequency of status labels assigned to the features simulated with differential abundance. Subset of simulations to TSS normalized datasets with main signal association strength of Spearman's rho = 0.5 and 16 additional covariates. a) exact percentages of associations labeled "AD" and b) frequency of all possible labels. Highest possible association between the main signal variable and the additional covariates is shown a) as color and b) faceted into three subpanels.

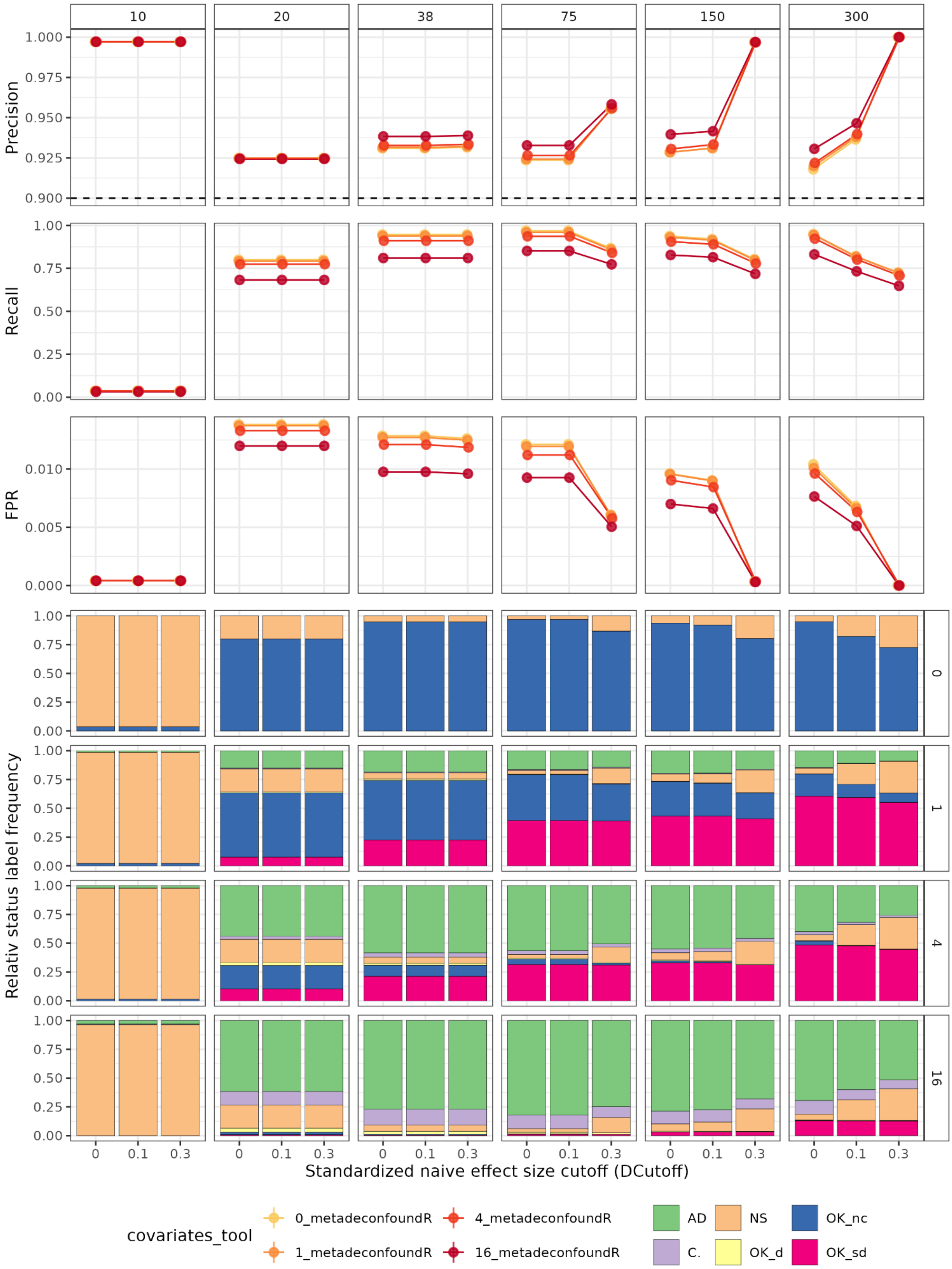


**Suppl. Figure 11: Applying more restrictive effect size filtering shows sample size-dependent effects.** Subset of simulations to TSS normalized datasets with main signal association strength of Spearman's rho = 0.5, and maximum possible covariate association strength rho = 0.99. The number of additional covariates is encoded as color in the dot plots and as separate rows in the bar plots.

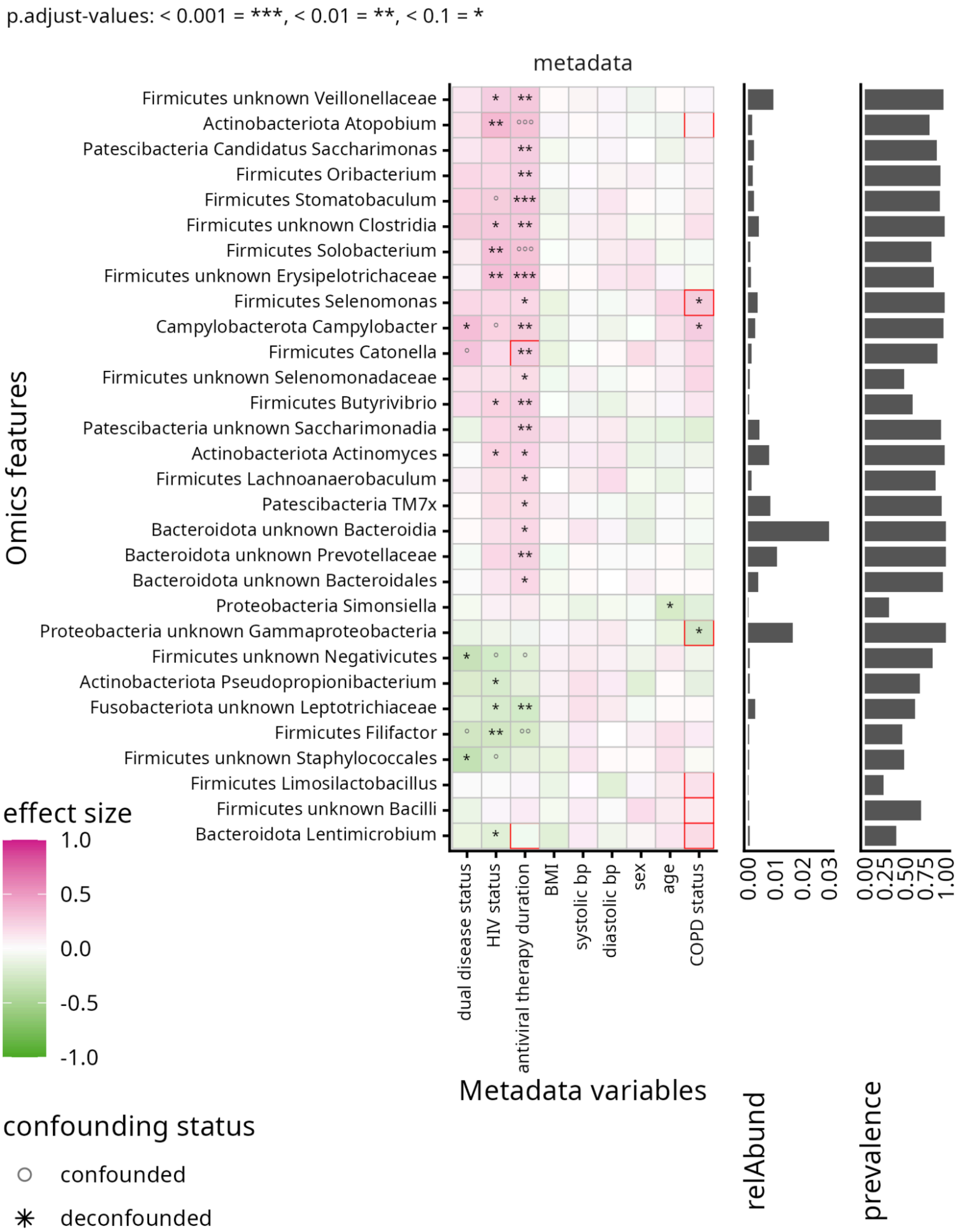


**Suppl. Figure 12: MetadeconfoundR and Maaslin2 output on real-world data with correlated covariates differs drastically.** Standard heatmap as produced by metadeconfoundR function BuildHeatmap(), expanded with bar plots showing mean relative abundance and prevalence per microbial feature. Tile fill color corresponds to naive standardized effect sizes, asterisks and circles show naive q-values as reported by metadeconfoundR. Tiles with red borders indicate associations reported by Maaslin2 when manually performing FDR correction per metadata variable.

**Supplementary Methods**

**Implementation: output of intermediate and final results**

The assigned labels are returned alongside the above-mentioned naive significance and effect size values either as a list of separate wide-format data frames or a single long format data frame. A nested list containing model objects of all models built during the post-hoc regression testing step can be added to the output optionally.

Instead of running the whole pipeline in a single run, intermediate output can be both returned by and read into MetaDeconfound(), separating the first “naive association testing” part from the subsequent post-hoc regression testing steps. A reduced data frame lacking the final confounder status labeling can be returned for further custom processing or storage. Alternatively, corrected p-values and effect sizes (either from previous metadeconfoundR runs, custom association testing pipelines, or otherwise generated from prior knowledge) can be supplied to metadeconfoundR in order to directly skip to the post-hoc regression testing steps. The convenience function ImportLongPrior() can be used to reformat data, like a list of known associations from literature, to fit the metadeconfoundR pipeline format requirements.

In case of collinearity of metadata variables, the standardized effect sizes returned by the main metadeconfoundR pipeline overestimate the actual effect of individual variables. To quantify the unique contribution of each metadata variable to the overall variance of a feature, partial effect sizes in the form of (normalized) partial $R^2$ values can be computed from the MetaDeconfound() output using the function GetPartialEfSizes(). For this, only the subset of metadata variables labeled by metadeconfoundR as having some significant association with the feature is included in one large model similar to the models described above. For each included metadata variable, an additional model without that variable as predictor is fitted and the absolute difference in $R^2$ of the entire model is reported as partial effect size. In addition, two “normalized” partial effect sizes are returned as well. For one, the partial $R^2$ of a metadata variable is divided by the $R^2$ of the full model (i.e. how much of the overall explainable variance of a feature is explained by the respective metadata variable). For the second normalization, the partial $R^2$ of a metadata variable is divided by (1 - $R^2$ of the smaller model) (i.e. how much of the not otherwise explainable variance of the feature can be explained by the respective metadata variable, or how much of its explanatory potential is reached.)

**Implementation: visualization of results for inspection and refinement of analysis**

Output of the MetaDeconfound() and GetPartialEfSizes() functions can be graphically summarized in a heatmap or cuneiform plot using the included BuildHeatmap() function (Fig. 1b). Each association is represented as a tile/triangle with effect size and direction encoded as color and significance represented by asterisks for non-confounded, significant associations, and circles for significant but confounded associations. Features (y-axis) and metadata variables (x-axis) by default are hierarchically clustered based on the effect size, so that, for example, groups of collinear variables can be identified easily by blocks of similarly shaded tiles/triangles. For more complex datasets where features are associated with a larger number of metadata variables, BuildConfounderMap() creates a circos plot for each feature showing confounding relationships between all metadata variables associated

with the respective feature (Fig. 1c). Note that these visualizations primarily support inspection of metadeconfoundR output and exploration of the data structure. Subsequent refinement of analyses may include the removal of collinear or redundant metadata variables and features, or re-evaluation of associations to determine whether apparent confounding effects reflect true confounding or potential mediation relationships. Such refinements, guided by domain knowledge and targeted statistical follow-up, are essential for meaningful interpretation and iterative improvement of metadeconfoundR analyses.

**Implementation: omics integration mode**

MetadeconfoundR can be supplied with a second dataframe of features that will be tested for associations with the first set of features alongside the supplied metadata. Here, multiple testing correction is applied over the number of both feature spaces combined, while p-values for the metadata are not corrected. In addition, associations between the two feature spaces are removed from the pool of potential confounders, so that feature-feature associations can only be confounded by metadata variables and not additional features. When graphically summarizing results of such an analysis run, the additional feature space and the metadata variables will be split in separate subpanels.
Feature-feature associations confounded by a metadata variable can be seen as candidates for further investigation of potential mediation effects.

**Implementation: parametric analysis**

Since metadeconfoundR's standard approach of rank transforming supplied omics features is not compatible with data representations intentionally encoding biologically relevant information in quantitative distances between observations, such as CLR transformation, an alternative parametric mode (clr_mode) was introduced. This mode uses Pearson correlation tests, t-tests and ANOVA for naive testing, Pearson correlation coefficient for naive standardized effect size calculation, and disables rank transforming features supplied to the nested models during confounder labelling.

**Benchmarking: Simulation design**

An additional simulation to generate a dataset with additive confounding effects was based on the script additiveConfounders.R available at the benchmarking repository (https://github.com/TillBirkner/metadeconfoundR_benchmarking). Here, two uncorrelated covariates jointly confounded the association of the main signal variable with five out of 200 simulated taxa in 100 samples. An additional five taxa were simulated to only have an association with the main signal variable. The dataset was analyzed with both MaAsLin2 and metadeconfoundR. MetadeconfoundR output was additionally fed to the accompanying function GetPartialEfSizes() and generated partial effect sizes were subsequently plotted.

**Benchmarking: performance evaluation**

MetadeconfoundR (v1.0.4) was run with default settings, switching to rawCount = TRUE or logistic = TRUE for rawCount and logistic regression modes. Features were filtered for prevalence > 4 prior to the function call. All non-confounded associations (OK_sd, OK_d, OK_nc, AD) with the main signal variable were considered significant at Qs < 0.1.

Additionally, a subset of simulations was re-analyzed with the metadeconfoundR effect size threshold DCutoff set to 0.1 or 0.3. Associations with effect sizes below these thresholds were labelled as  not significant and thus filtered.
To provide practical guidance to users, the prevalence of the different status labels assigned by metadeconfoundR was also collected and summarized.

MaAsLin2 (v1.20.0) was run using the linear model (LM) framework with Benjamini-Hochberg multiple testing correction and a significance cutoff of q < 0.1. Features were analyzed without internal normalization and using log transformation in all cases with the exception of  CLR transformed data. Features were only filtered for prevalence (min_variance = 0, min_abundance = 0, min_prevalence = 4/sample_size). The main signal variable and all simulated covariates (if present) were included as fixed effects. Associations with the main signal variable were considered significant at q_val < 0.1 based on the output file "all_results.tsv". Since q_val is generated using global multiple-testing correction,  the raw p_val values were corrected separately per metadata variable as an alternative approach that more closely resembles the FDR correction strategy performed in metadeconfoundR.

Computational resources were monitored by logging CPU usage with base R function proc.time() and memory usage with the peakRAM() function from the peakRAM package.
